# Publisher Correction: Accelerating functional gene discovery in osteoarthritis

**DOI:** 10.1038/s41467-021-23768-8

**Published:** 2021-05-28

**Authors:** Natalie C. Butterfield, Katherine F. Curry, Julia Steinberg, Hannah Dewhurst, Davide Komla-Ebri, Naila S. Mannan, Anne-Tounsia Adoum, Victoria D. Leitch, John G. Logan, Julian A. Waung, Elena Ghirardello, Lorraine Southam, Scott E. Youlten, J. Mark Wilkinson, Elizabeth A. McAninch, Valerie E. Vancollie, Fiona Kussy, Jacqueline K. White, Christopher J. Lelliott, David J. Adams, Richard Jacques, Antonio C. Bianco, Alan Boyde, Eleftheria Zeggini, Peter I. Croucher, Graham R. Williams, J. H. Duncan Bassett

**Affiliations:** 1 Molecular Endocrinology Laboratory, Department of Metabolism, Digestion and Reproduction, Imperial College London, London, W12 0NN UK; 2 Institute of Translational Genomics, Helmholtz Zentrum München – German Research Center for Environmental Health, 85764 Neuherberg, Germany; 3 Wellcome Trust Sanger Institute, Hinxton, Cambridge, CB10 1SA UK; 4 Cancer Council NSW, Sydney, NSW 2000 Australia; 5 The Garvan Institute of Medical Research and St. Vincent’s Clinical School, University of New South Wales Medicine, Sydney, NSW 2010 Australia; 6 Department of Oncology and Metabolism, University of Sheffield, Sheffield, S10 2RX UK; 7 Centre for Integrated Research into Musculoskeletal Ageing and Sheffield Healthy Lifespan Institute, University of Sheffield, Sheffield, S10 2TN UK; 8 Division of Endocrinology and Metabolism, Rush University Medical Center, Chicago, IL 60612 USA; 9 The Jackson Laboratory, Bar Harbor, ME 04609 USA; 10 School of Health and Related Research (ScHARR), University of Sheffield, Sheffield, S1 4DA UK; 11 Section of Adult and Pediatric Endocrinology, Diabetes & Metabolism, Department of Medicine, University of Chicago, Chicago, IL 60637 USA; 12 Dental Physical Sciences, Queen Mary University of London, Mile End Road, London, E1 4NS UK

**Keywords:** Bone, Cartilage

Correction to: *Nature Communications* 10.1038/s41467-020-20761-5, published online 20 January 2021.

The original version of this Article contained private information in the Data Availability statement, which was used by reviewers to access Proteomics datasets. This information has now been removed from both the PDF and HTML versions of this article.

