# Supplementary material for: Accelerating functional gene discovery in osteoarthritis
Source: Nat Commun. 2021 Jan 20;12:467. doi: 10.1038/s41467-020-20761-5 (PMC7817695; doi:10.1038/s41467-020-20761-5)
Supplement: Supplementary file 1 — Supplementary Information [file 41467_2020_20761_MOESM1_ESM.pdf]

Accelerating functional gene discovery in osteoarthritis

Natalie C. Butterfield[1], Katherine F. Curry[1], Julia Steinberg[2,3,4], Hannah Dewhurst[1], Davide Komla-Ebri[1], Naila S. Mannan[1], Anne-Tounsia Adoum[1], Victoria D. Leitch[1], John G. Logan[1], Julian A. Waung[1], Elena Ghirardello[1], Lorraine Southam[2,3], Scott E. Youlten[5], J, Mark Wilkinson[6,7], Elizabeth A. McAninch[8], Valerie E. Vancollie[3], Fiona Kussy[3], Jacqueline K. White[3,9], Christopher J. Lelliott[3], David J. Adams[3], Richard Jacques[10], Antonio C. Bianco[11], Alan Boyde[12], Eleftheria Zeggini[2,3], Peter I. Croucher[5], Graham R. Williams[1,13*] and J. H. Duncan Bassett[1,13*]

[1]Molecular Endocrinology Laboratory, Department of Metabolism, Digestion and Reproduction, Imperial College London, London W12 0NN, UK.

[2]Institute of Translational Genomics, Helmholtz Zentrum München – German Research Center for Environmental Health, 85764 Neuherberg, Germany

[3]Wellcome Trust Sanger Institute, Hinxton, Cambridge CB10 1SA, UK

[4]Cancer Council NSW, Sydney, New South Wales 2000, Australia

[5]The Garvan Institute of Medical Research and St. Vincent's Clinical School, University of New South Wales Medicine, Sydney, New South Wales 2010, Australia

[6]Department of Oncology and Metabolism, University of Sheffield, Sheffield S10 2RX, UK

[7]Centre for Integrated Research into Musculoskeletal Ageing and Sheffield Healthy Lifespan Institute, University of Sheffield, Sheffield S10 2TN, UK

[8]Division of Endocrinology and Metabolism, Rush University Medical Center, Chicago, IL 60612, USA

[9]The Jackson Laboratory, Bar Harbor, ME 04609, USA

[10]School of Health and Related Research (ScHARR), University of Sheffield, Sheffield S1 4DA, UK

[11]Section of Adult and Pediatric Endocrinology, Diabetes & Metabolism, Department of Medicine, University of Chicago, Chicago, IL 60637, USA

[12]Dental Physical Sciences, Queen Mary University of London, Mile End Road, London E1 4NS, UK

[13]These authors contributed equally

*Corresponding authors

Supplementary Information Contents

Supplementary Figures

1. Reference range data for joint phenotype parameters
2. Joint abnormalities following destabilization of the medial meniscus (DMM) surgery and severe early onset osteoarthritis in *Pitx1*$^{+/-}$ mice
3. Synovitis and osteophyte formation in destabilization of the medial meniscus (DMM)-operated mice
4. Validation of imaging methods
5. Early onset osteoarthritis in *Bhlhe40*$^{-/-}$ and *Sh3pb4*$^{-/-}$ mutant mice
6. Early onset osteoarthritis in mice with deletion of genes differentially expressed in human osteoarthritis cartilage
7. Age-related joint degeneration
8. Mice with a *Dio2*^{Ala92} polymorphism are protected from osteoarthritis
9. Origins of Bone and Cartilage Disease (OBCD) rapid-throughput joint phenotyping compared to Osteoarthritis Research Society International (OARSI) histological scoring
10. Validation of iodine contrast-enhanced µCT (ICEµCT)

Supplementary Methods

Supplementary References

Supplementary Figure 1

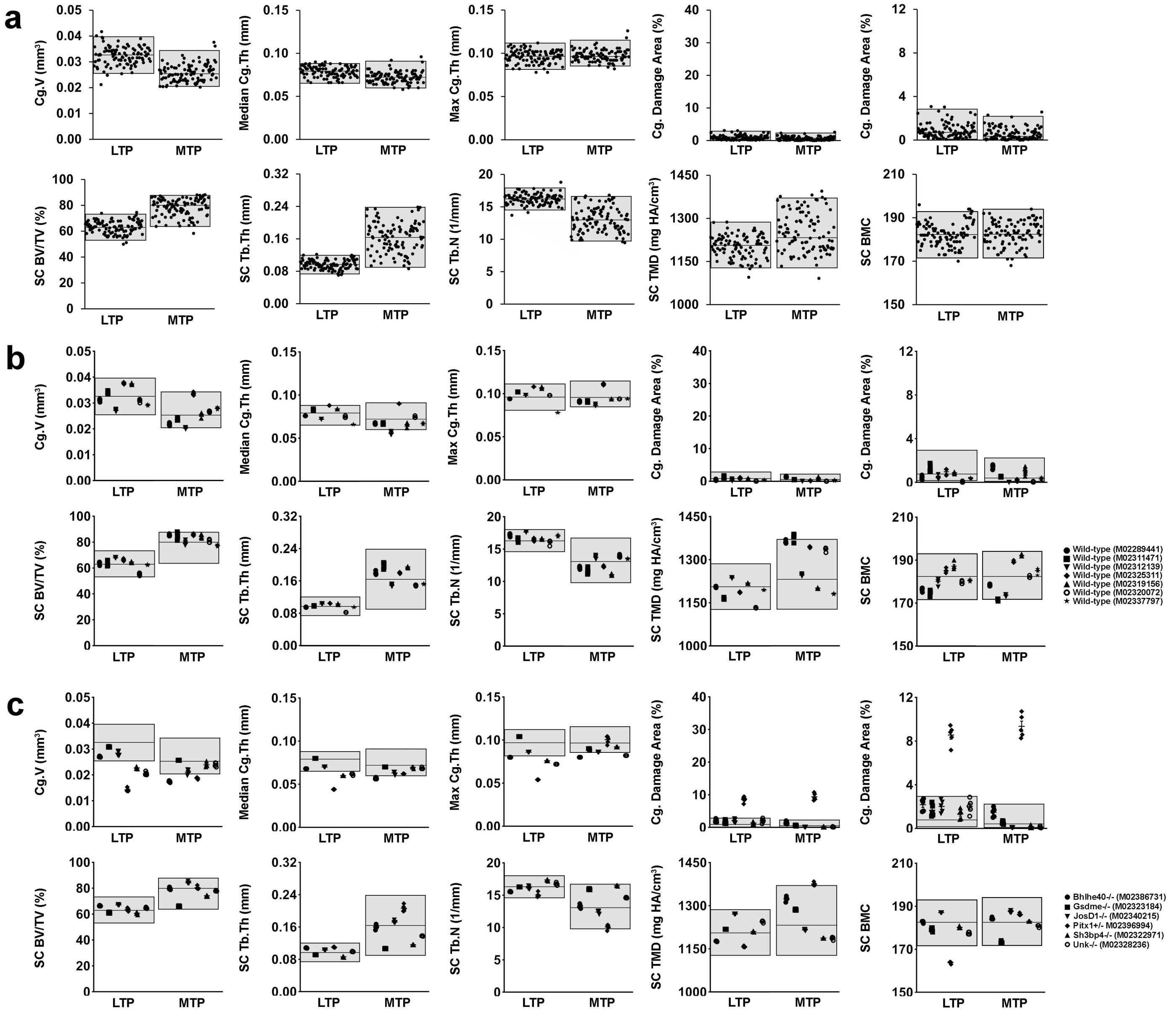

Reference range data for joint phenotype parameters

a. Graphs showing articular cartilage (articular cartilage volume (Cg.V), median articular cartilage thickness (Median Cg.Th), maximum articular cartilage thickness (Max Cg.Th), Cg. damage area) and subchondral bone (subchondral bone volume per tissue volume (SC BV/TV), SC trabecular thickness (SC Tb.Th), SC trabecular number (SC Tb.N), SC tissue mineral density (SC TMD), SC bone mineral content (SC BMC)) parameters in the lateral and medial tibial plateaux (LTP, MTP) of 16-week-old male WT mice (n=100). Black dots: individual mice.

b. Repeatability of measurement for each parameter determined by five repeat analyses of seven samples obtained from individual 16-week-old wild-type mice. Black symbols are repeat measures for each sample, with mean ± standard error of the mean.

c. Repeatability of measurement for each parameter determined by five repeat analyses of six samples obtained from individual 16-week-old mutant mice representing the full spectrum of phenotype severity. Black symbols are repeat measures for each sample with mean ± standard error of the mean.

In ***a-c***, grey boxes are reference ranges derived from 100 wild-type samples. For normally distributed parameters [Cg.V (LTP), Max Cg.Th (LTP), SC-BV/TV (LTP), SC Tb.Th (LTP, MTP), SC Tb.N (LTP), SC TMD (LTP), SC BMC (LTP, MTP)], reference range is 2 standard deviations above and below the mean (black line). For non-normally distributed parameters [Cg.V (MTP), Median Cg.Th (LTP, MTP), Max Cg.Th (MTP), Cg. Damage Area (LTP, MTP), SC-BV/TV (MTP), SC Tb.N (MTP), SC TMD (MTP)], reference range is the 2.5th-97.5th percentile, and black line is the median. mm; millimeters, mg HA/cm^3; milligrams of hydroxyapatite/cubic centimeter. Source data are provided as a Source Data file.

Supplementary Figure 2

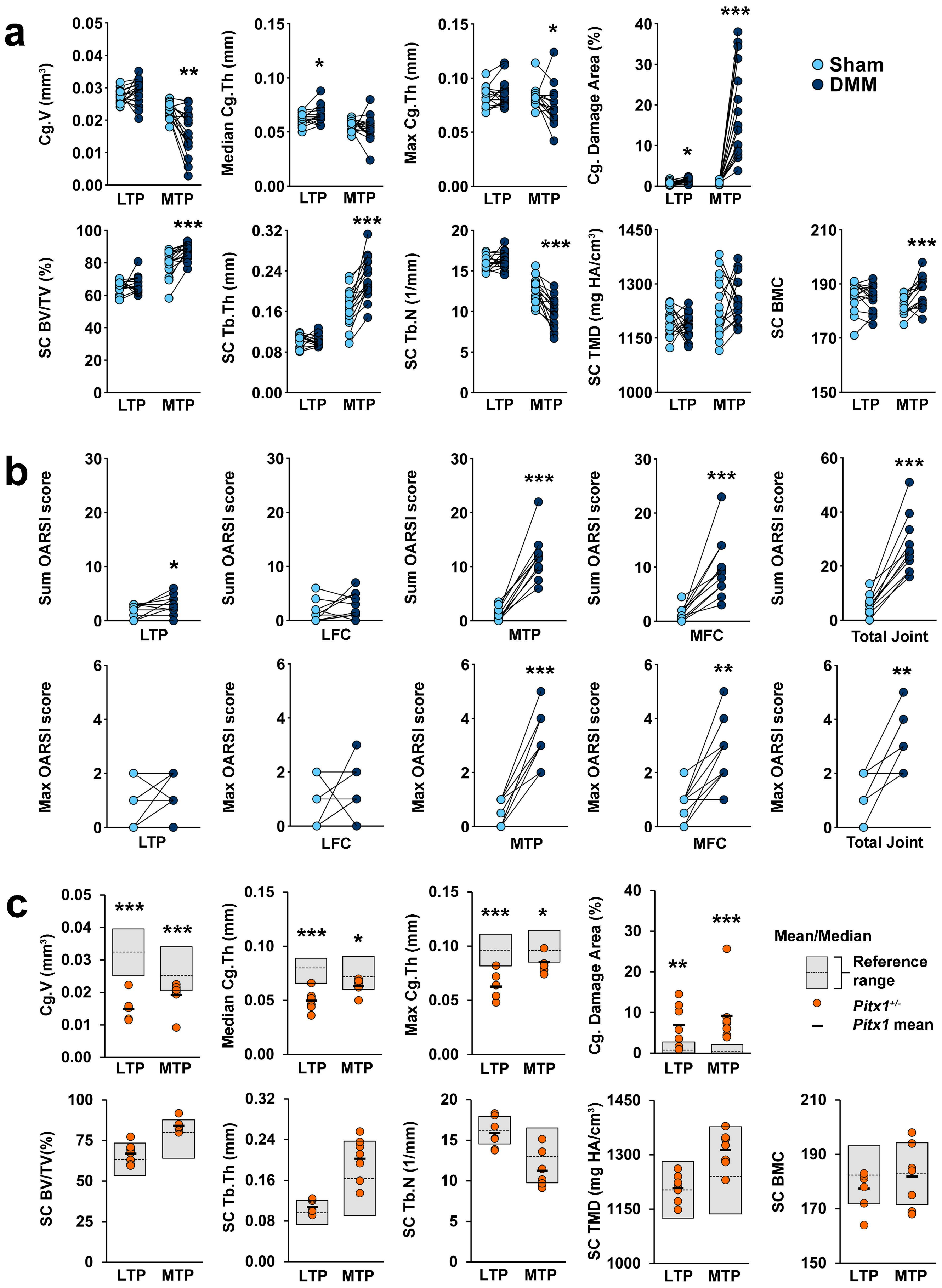

Supplementary Figure 2

Joint abnormalities following destabilization of the medial meniscus (DMM) surgery and severe early onset osteoarthritis in Pitx1$^{+/-}$ mice

a. Graphs showing articular cartilage (articular cartilage volume (Cg.V), median articular cartilage thickness (Median Cg.Th), maximum articular cartilage thickness (Max Cg.Th), Cg. damage area) and subchondral bone (subchondral bone volume per tissue volume (SC BV/TV), SC trabecular thickness (SC Tb.Th), SC trabecular number (SC Tb.N), SC tissue mineral density (SC TMD), SC bone mineral content (SC BMC)) parameters in the lateral and medial tibial plateaux (LTP, MTP) of sham and DMM-operated knees of wild type (WT) mice 12 weeks after surgery (n=16). Paired data (sham versus DMM) are shown for each mouse. **P*<0.05, ***P*<0.01, 2-tailed Wilcoxon matched pairs signed rank test for Cg.V (MTP), Median Cg.Th (LTP, MTP), Max Cg.Th (MTP), Cg. Damage Area (LTP, MTP), SC-BV/TV (MTP), SC Tb.N (MTP) and SC TMD (MTP), or 2-tailed paired *t*-test for Cg.V (LTP), Max Cg.Th (LTP), SC-BV/TV (LTP), SC Tb.Th (LTP, MTP), SC Tb.N (LTP), SC TMD (LTP), SC BMC (LTP, MTP). Cg.V (MTP): *P*=0.001312, Median Cg.Th (LTP): *P*=0.021729, Max. Cg.Th (MTP): *P*=0.017334, Cg. Damage Area (LTP): *P*=0.013092, (MTP): *P*=0.000031, SC BV/TV (MTP): *P*=0.000061, SC Tb.Th (MTP): *P*=0.000013, SC Tb.N (MTP): *P*=0.000031, SC BMC (MTP): *P*=0.000477.

b. Graphs showing Osteoarthritis Research Society International (OARSI) histological scores in sham and DMM-operated knees of WT mice 12 weeks after surgery (n=11). Parameters include the summed and maximum scores on the LTP, lateral femoral condyle (LFC), MTP, medial femoral condyle (MFC), and combined values for the total joint. **P*<0.05, ***P*<0.01, 2-tailed Wilcoxon matched pairs signed rank test. Sum OARSI scores: LTP: *P*=0.0469, MTP: *P*=0.000977, MFC: *P*=0.000977, total joint: *P*=0.000977. Max. OARSI scores: MTP: *P*=0.000977, MFC: *P*=0.001953, total joint: *P*=0.001953.

c. Graphs showing articular cartilage and subchondral bone parameters in the LTP and MTP of 16-week-old *Pitx1*$^{+/-}$ mice (n=7). Orange circles: individual mutant samples, black horizontal lines: sample mean. Grey boxes: reference ranges derived from 100 wild-type samples. For normally distributed parameters [Cg.V (LTP), Max Cg.Th (LTP), SC-BV/TV (LTP), SC Tb.Th (LTP, MTP), SC Tb.N (LTP), SC TMD (LTP), SC BMC (LTP, MTP)], reference range is 2 standard deviations above and below the mean (dashed line). For non-normally distributed parameters [Cg.V (MTP), Median Cg.Th (LTP, MTP), Max Cg.Th (MTP), Cg. Damage Area (LTP, MTP), SC-BV/TV (MTP), SC Tb.N (MTP) SC TMD (MTP)], reference range is the 2.5th-97.5th percentile, and dashed line is the median. **P*<0.00568, ***P*<0.001, ****P*<0.0001, Bonferroni-corrected 2-tailed Wilcoxon rank sum test. Cg.V (LTP): *P*=0.00001, (MTP): *P*=0.00008, Median Cg.Th (LTP): *P*=0.00001, (MTP): *P*=0.00307 Max. Cg.Th (LTP): *P*=0.00001, (MTP): *P*=0.00319, Cg. Damage Area (LTP): *P*=0.00014 (MTP): *P*=0.00001. mm; millimeters, mg HA/cm^3; milligrams of hydroxyapatite/cubic centimeter. Source data are provided as a Source Data file.

Supplementary Figure 3

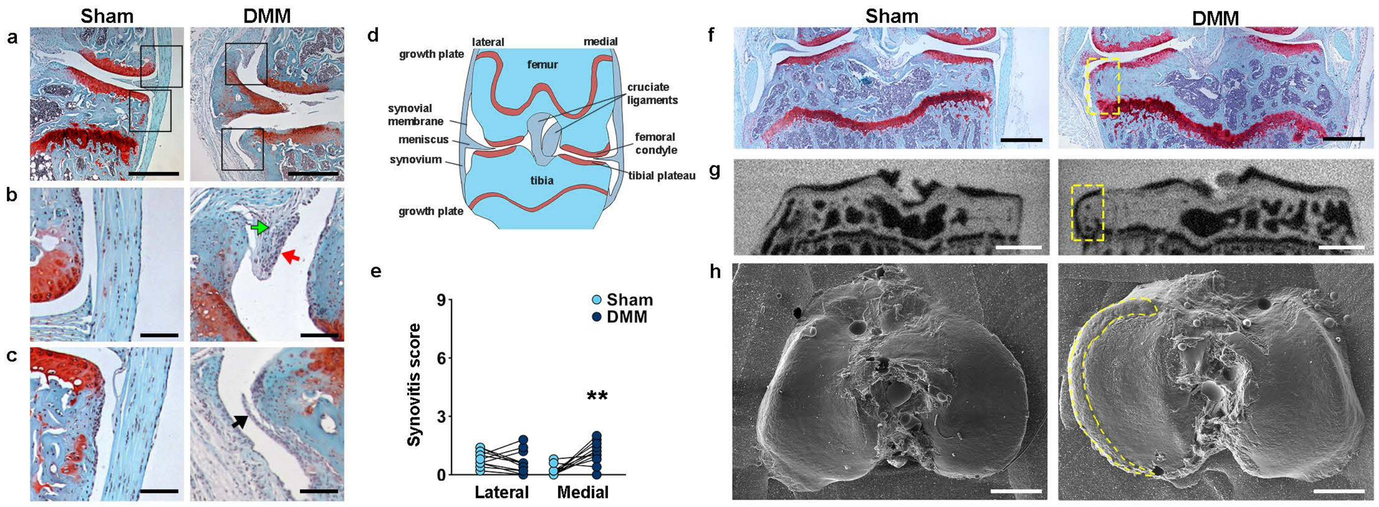

Synovitis and osteophyte formation in destabilization of the medial meniscus (DMM)-operated mice

a. Example Safranin O/Fast green-stained coronal sections from sham and DMM-operated joints demonstrating the signs of synovitis in the operated knee. Black boxes indicate regions of interest shown in ***b*** and ***c***.

b. Synovial hyperplasia (red arrow, 10/11 knees) and sub-synovial inflammation with new blood vessel formation (green arrow, 8/11 knees) are shown in the DMM-operated knee.

c. Pannus formation (black arrow, 1/11 knee) in the DMM-operated knee.

d. Schematic representation of the mouse knee joint, red; cartilage, blue; bone, grey; soft tissue

e. Mean synovitis scores in the lateral and medial compartments of sham and DMM-operated knees (n=11, ***P*= 0.0059, 2-tailed Wilcoxon matched-pairs signed rank test). Source data are provided as a Source Data file.

f. Safranin O/Fast green-stained coronal sections from sham and DMM-operated joints showing osteophyte formation (yellow box).

g. Iodine contrast-enhanced µCT (ICEµCT) images showing osteophyte formation (yellow box).

h. Joint surface replica (JSR) images showing osteophyte formation outlined in yellow. Scale bars = 500µm (panels a, f, g, h), and 100µm (panels b, c).

Supplementary Figure 4

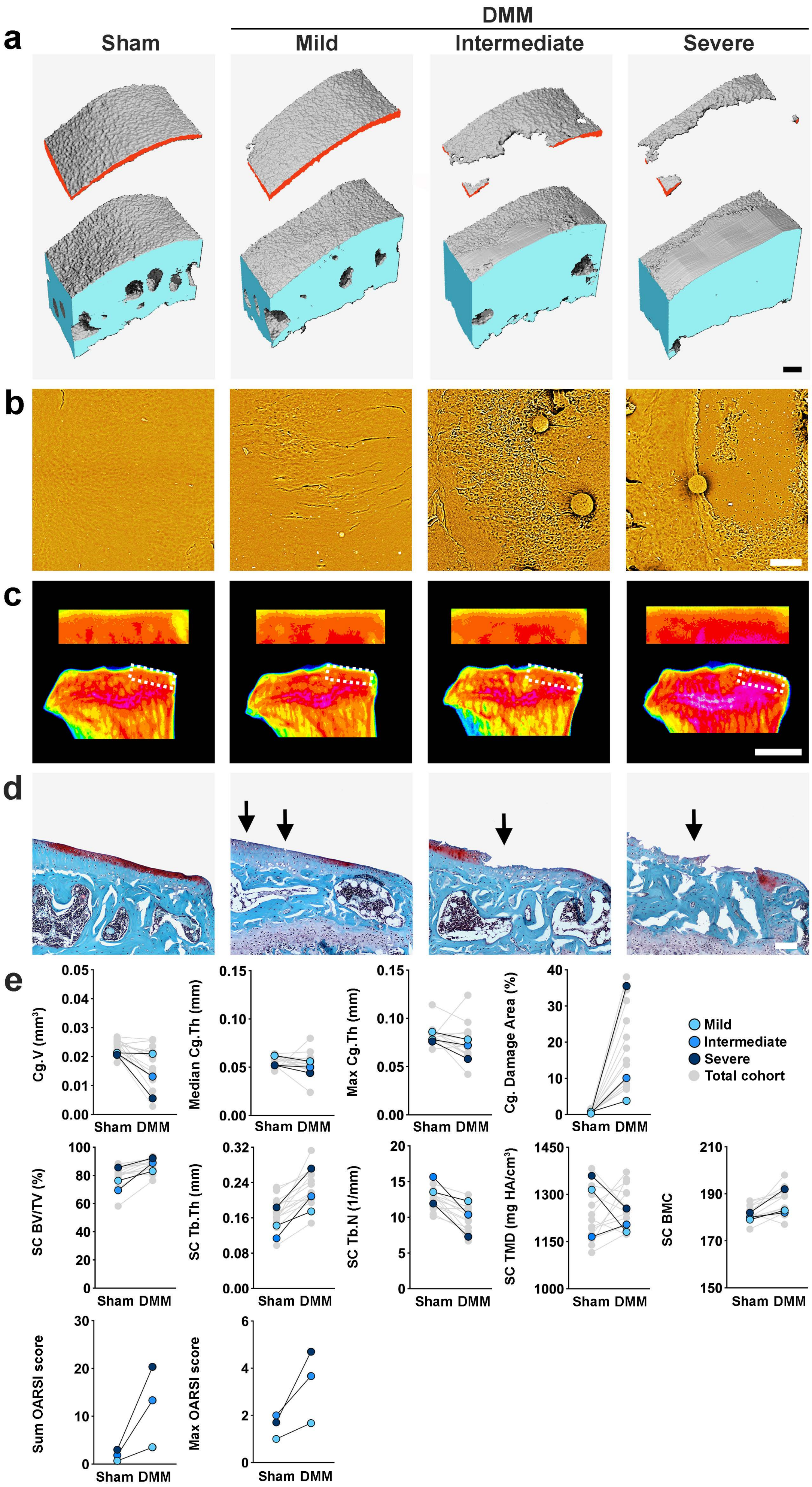

Validation of imaging methods

a. Iodine contrast enhanced μCT images of medial tibial plateau articular cartilage (red) and subchondral bone (blue) from 22-week-old wild type (WT) male mice 12 weeks after sham operation or following destabilization of the medial meniscus (DMM) surgery that resulted in either mild, intermediate or severe osteoarthritis (OA).

b. Back-scattered electron scanning-electron microscopy images of medial tibial plateau joint surface replicas from sham and DMM-operated WT mice with mild, intermediate or severe OA.

c. X-ray microradiography images of proximal tibia and the medial tibial plateau subchondral bone region of interest (dashed box) from sham and DMM-operated WT mice with mild, intermediate or severe OA. Greyscale images are pseudocoloured according to a 16 colour look-up table in which low bone mineral content (BMC) is yellow and high BMC is pink.

d. Coronal sections of medial tibial plateaux stained with Safranin-O/Fast green from three sham and three DMM-operated WT mice with mild, intermediate or severe OA (Sections assessed at 5 levels through each knee joint). Arrows indicate areas of cartilage damage. Scale bars = 100μm (a, b, d) and 1mm (c).

e. Graphs showing articular cartilage (articular cartilage volume (Cg.V), median articular cartilage thickness (Median Cg.Th), maximum articular cartilage thickness (Max Cg.Th) and Cg. damage area) and subchondral bone (subchondral bone volume per tissue volume (SC BV/TV), SC trabecular thickness (SC Tb.Th), SC trabecular number (SC Tb.N), SC tissue mineral density (SC TMD), SC bone mineral content (SC BMC)) parameters together with summed and maximum Osteoarthritis Research Society International (OARSI) histological scores on the medial tibial plateaux of sham and DMM-operated knees from three mice with mild, intermediate and severe OA. Results from the entire cohort in grey, with mild, intermediate and severe examples coloured according to key. mm; millimeters, mg HA/cm^3; milligrams of hydroxyapatite/cubic centimeter. Source data are provided as a Source Data file.

Supplementary Figure 5

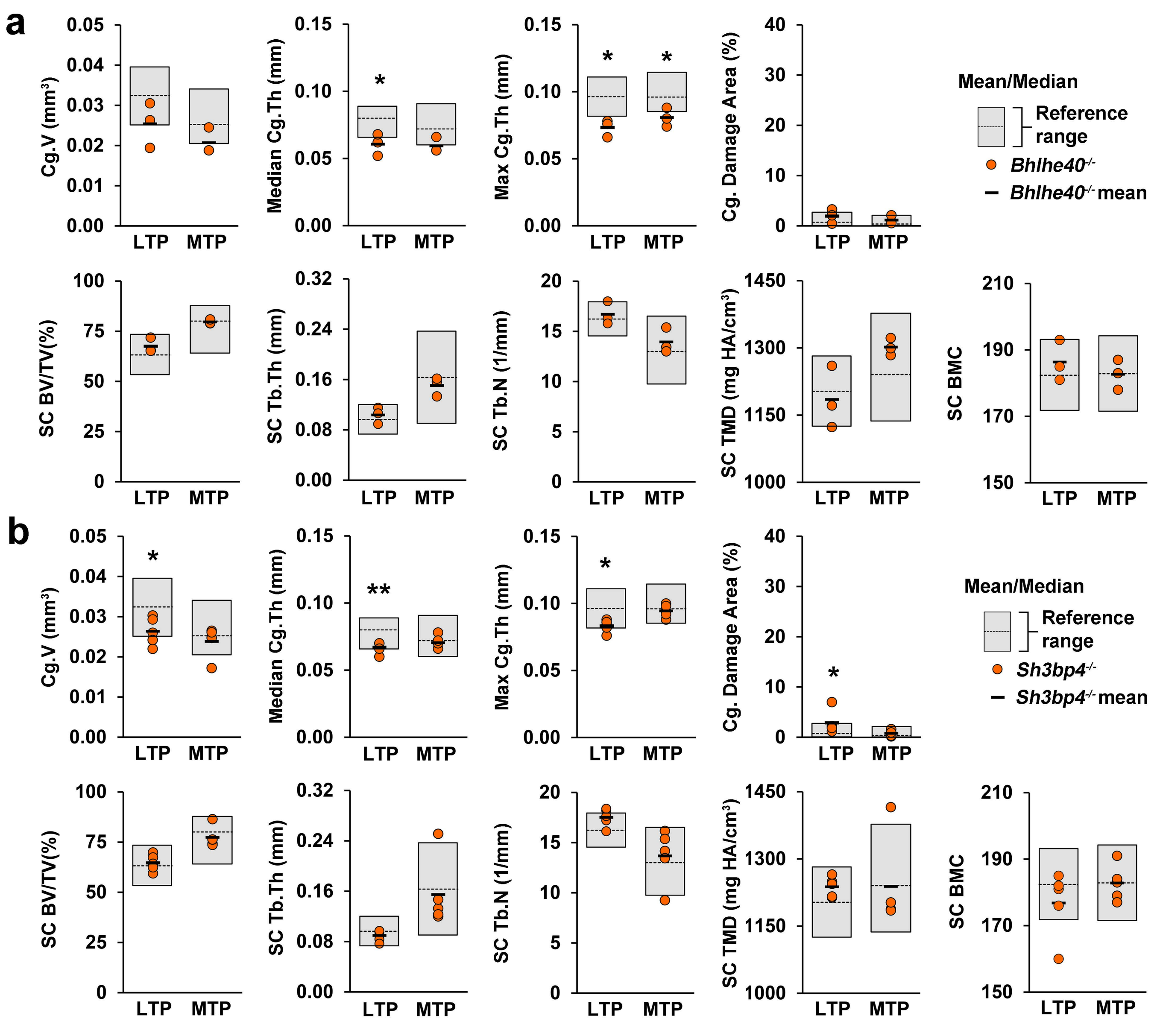

Early onset osteoarthritis in Bhlhe40$^{-/-}$ and Sh3pb4$^{-/-}$ mutant mice

a. Graphs showing articular cartilage (articular cartilage volume (Cg.V), median articular cartilage thickness (Median Cg.Th), maximum articular cartilage thickness (Max Cg.Th) and Cg. damage area) and subchondral bone (subchondral bone volume per tissue volume (SC BV/TV), SC trabecular thickness (SC Tb.Th), SC trabecular number (SC Tb.N), SC tissue mineral density (SC TMD), SC bone mineral content (SC BMC)) parameters in the lateral and medial tibial plateaux (LTP, MTP) from 16-week-old *Bhlhe40*$^{-/-}$ mice (n=3).

b. Graphs showing articular cartilage and subchondral bone parameters in the LTP and MTP from 16-week-old *Sh3pb4*$^{-/-}$ mice (n=5). Orange circles: individual mutant samples, black horizontal lines: sample mean. Grey boxes: reference ranges derived from 100 wild-type samples. For normally distributed parameters [Cg.V (LTP), Max Cg.Th (LTP), SC-BV/TV (LTP), SC Tb.Th (LTP, MTP), SC Tb.N (LTP), SC TMD (LTP), SC BMC (LTP, MTP)], reference range is 2 standard deviations above and below the mean (dashed line). For non-normally distributed parameters [Cg.V (MTP), Median Cg.Th (LTP, MTP), Max Cg.Th (MTP), Cg. Damage Area (LTP, MTP), SC-BV/TV (MTP), SC Tb.N (MTP), SC TMD (MTP)], reference range is the 2.5th-97.5th percentile, and dashed line is the median. **P*<0.00568, ***P*<0.001, Bonferroni-corrected 2-tailed Wilcoxon rank sum test. *Bhlhe40*$^{-/-}$: Median Cg.Th (LTP): *P*=0.00469, Max. Cg.Th (LTP): *P*=0.00347, Max. Cg.Th (MTP): *P*=0.00498. *Sh3bp4*$^{-/-}$: Cg.V (LTP): *P*=0.00311, Median Cg.Th (LTP): *P*=0.00081. Max. Cg.Th (LTP): *P*=0.00133, Cg. Damage Area (LTP): *P*=0.00282. mm; millimeters, mg HA/cm^3; milligrams of hydroxyapatite/cubic centimeter. Source data are provided as a Source Data file.

Supplementary Figure 6

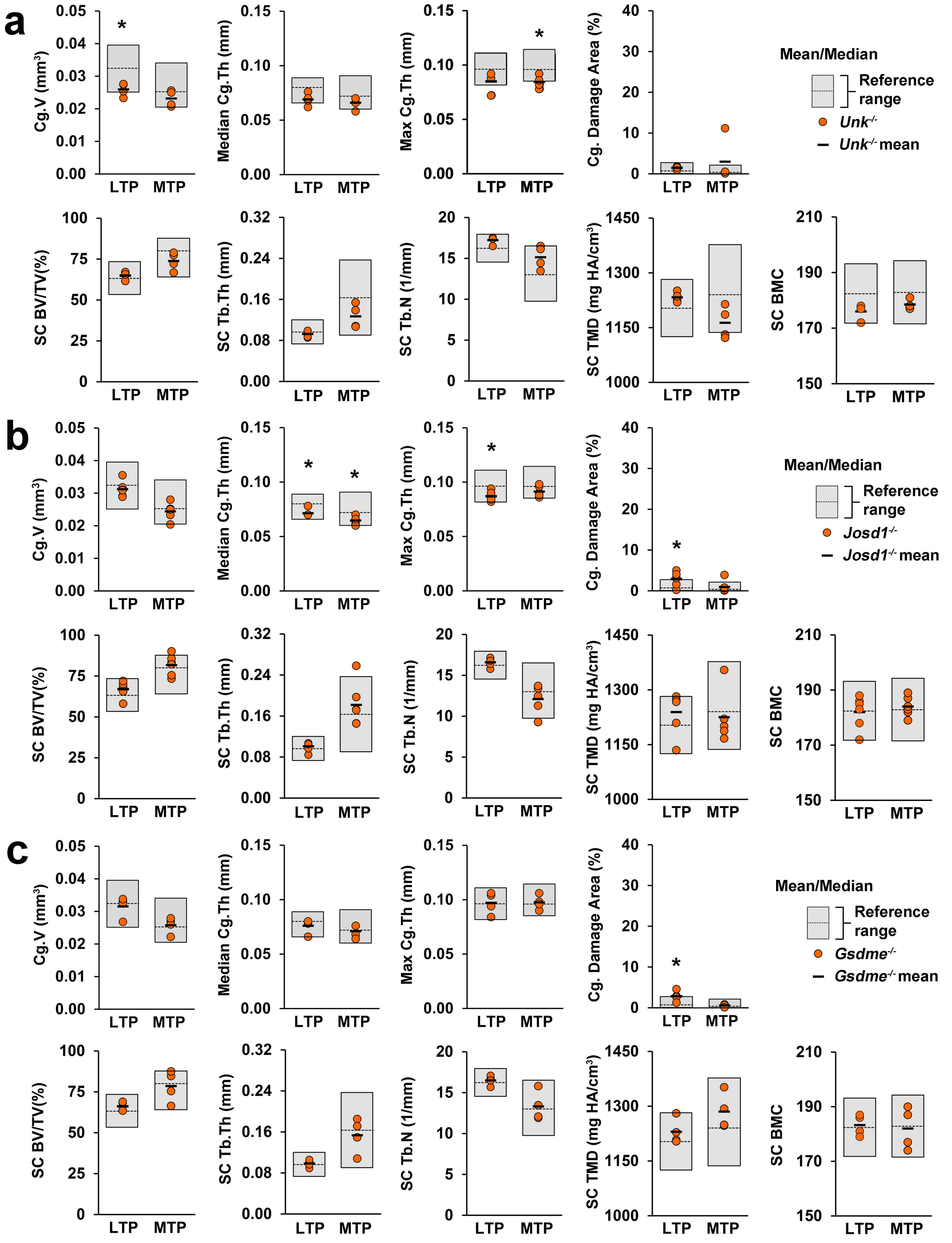

Early onset osteoarthritis in mice with deletion of genes differentially expressed in human osteoarthritis cartilage

a. Graphs showing articular cartilage (articular cartilage volume (Cg.V), median articular cartilage thickness (Median Cg.Th), maximum Cg.Th (Max Cg.Th), Cg. damage area) and subchondral bone (subchondral bone volume per tissue volume (SC BV/TV), SC trabecular thickness (SC Tb.Th), SC trabecular number (SC Tb.N), SC tissue mineral density (SC TMD), SC bone mineral content (SC BMC)) parameters in the lateral and medial tibial plateaux (LTP, MTP) of 16-week-old *Unk* $^{-/-}$ mice (n=4).

b. Graphs showing articular cartilage and subchondral bone parameters in the LTP and MTP of 16-week-old *Josd1* $^{-/-}$ mice (n=6).

c. Graphs showing articular cartilage and subchondral bone parameters in the LTP and MTP of 16-week-old *Gsdme* $^{-/-}$ mice (n=4). Orange circles: individual mutant samples, black horizontal lines: sample mean. Grey boxes: reference ranges derived from 100 wild-type samples. For normally distributed parameters [Cg.V (LTP), Max Cg.Th (LTP), SC-BV/TV (LTP), SC Tb.Th (LTP, MTP), SC Tb.N (LTP), SC TMD (LTP), SC BMC (LTP, MTP)], reference range is 2 standard deviations above and below the mean (dashed line). For non-normally distributed parameters [Cg.V (MTP), Median Cg.Th (LTP, MTP), Max Cg.Th (MTP), Cg. Damage Area (LTP, MTP), SC-BV/TV (MTP), SC Tb.N (MTP), SC TMD (MTP)], reference range is the 2.5th-97.5th percentile, and dashed line is the median. *P<0.00568, Bonferroni-corrected 2-tailed Wilcoxon rank sum test. *Unk* $^{-/-}$: Cg.V (LTP): P=0.00215, Max Cg.Th (MTP): P=0.00295. *Josd* $^{-/-}$: Median Cg.Th (LTP): P=0.00344, (MTP): P=0.00487, Max Cg.Th (LTP): P=0.00362, Cg. Damage area (LTP): P=0.00563. *Gsdme* $^{-/-}$: Cg. Damage Area (LTP): P=0.00355. mm; millimeters, mg HA/cm^3; milligrams of hydroxyapatite/cubic centimeter. Source data are provided as a Source Data file.

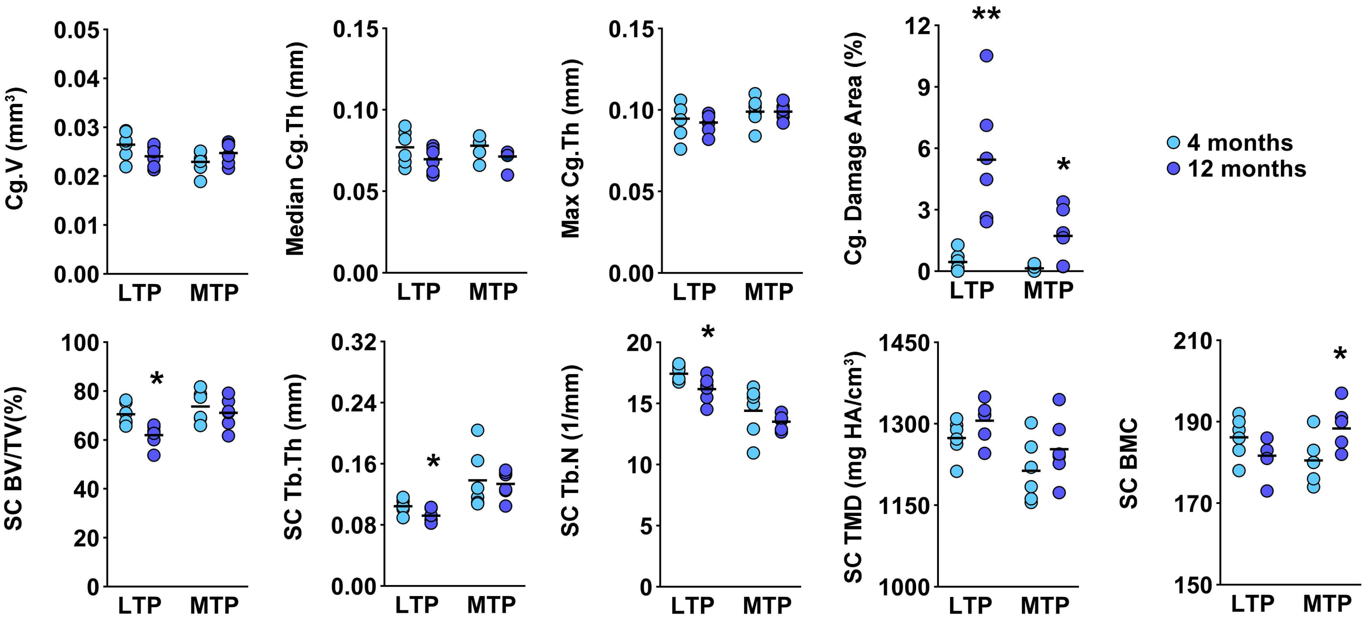

Age-related joint degeneration

Graphs showing articular cartilage (articular cartilage volume (Cg.V), median articular cartilage thickness (Median Cg.Th), maximum Cg.Th (Max Cg.Th), Cg. damage area) and subchondral bone (subchondral bone volume per tissue volume (SC BV/TV), SC trabecular thickness (SC Tb.Th), SC trabecular number (SC Tb.N), SC tissue mineral density (SC TMD), SC bone mineral content (SC BMC)) parameters in the lateral and medial tibial plateaux (LTP, MTP) of 4-month-old (n=6) and 12-month-old (n=6) WT mice. *P<0.05, **P<0.01, 2-tailed Wilcoxon rank sum test for Cg.V (MTP), Median Cg.Th (LTP, MTP), Max Cg.Th (MTP), Cg. Damage Area (LTP, MTP), SC-BV/TV (MTP), SC Tb.N (MTP) and SC TMD (MTP), or 2-tailed t-test for Cg.V (LTP), Max Cg.Th (LTP), SC-BV/TV (LTP), SC Tb.Th (LTP, MTP), SC Tb.N (LTP), SC TMD (LTP) and SC BMC (LTP, MTP). Cg. Damage Area (LTP): P=0.002, (MTP): P=0.026, SC BV/TV (LTP): P=0.008, SC Tb.Th (LTP): P=0.039, SC Tb.N (LTP): P=0.035, SC BMC (MTP): P=0.034. mm; millimeters, mg HA/cm^3; milligrams of hydroxyapatite/cubic centimeter. Source data are provided as a Source Data file.

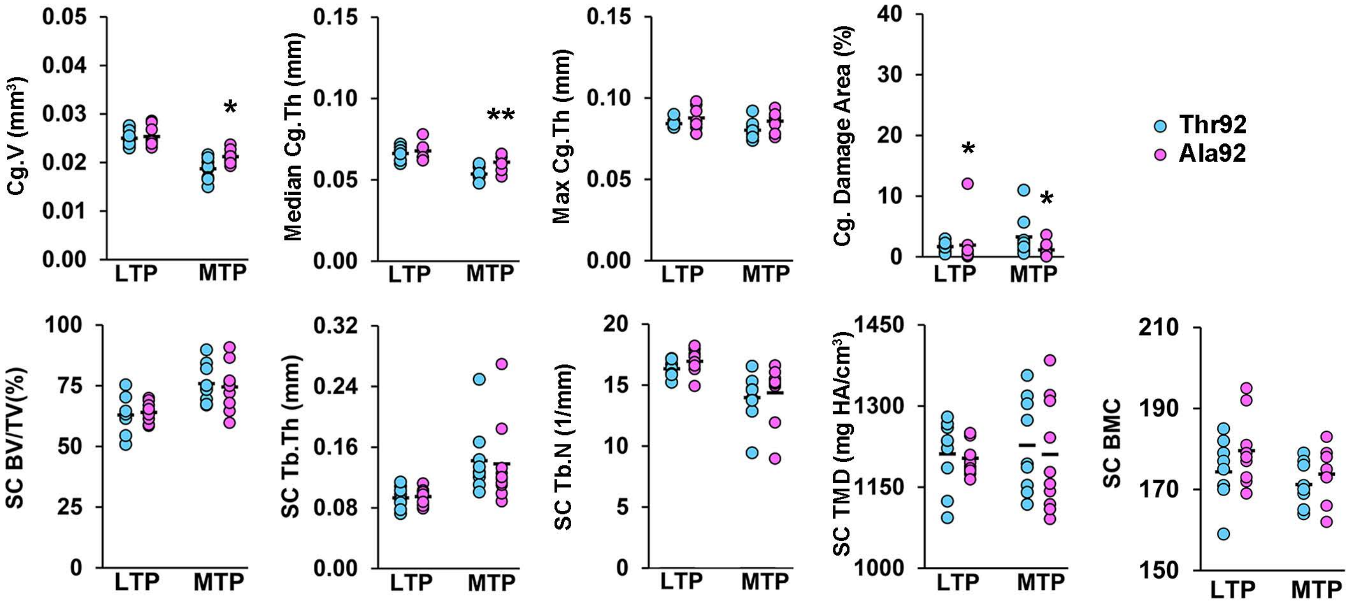

Mice with a Dio2^{Ala92} polymorphism are protected from osteoarthritis

Graphs showing articular cartilage (articular cartilage volume (Cg.V), median articular cartilage thickness (Median Cg.Th), maximum articular cartilage thickness (Max Cg.Th), Cg. damage area) and subchondral bone (subchondral bone volume per tissue volume (SC BV/TV), SC trabecular thickness (SC Tb.Th), SC trabecular number (SC Tb.N), SC tissue mineral density (SC TMD), SC bone mineral content (SC BMC)) parameters in the lateral and medial tibial plateaux (LTP, MTP) from 16-week-old *Dio2^{Thr92}* (n=9) and *Dio2^{Ala92}* (n=10) mice. *P<0.05, **P<0.01, 2-tailed Wilcoxon rank sum test for Cg.V (MTP), Median Cg.Th (LTP, MTP), Max Cg.Th (MTP), Cg. Damage Area (LTP, MTP), SC-BV/TV (MTP), SC Tb.N (MTP) and SC TMD (MTP), or 2-tailed *t*-test for Cg.V (LTP), Max Cg.Th (LTP), SC-BV/TV (LTP), SC Tb.Th (LTP, MTP), SC Tb.N (LTP), SC TMD (LTP), SC BMC (LTP, MTP). Cg.V (MTP): P=0.019, Median Cg.Th (MTP): P=0.006, Cg. Damage Area (LTP): P=0.043, (MTP): P=0.016. mm; millimeters, mg HA/cm^3; milligrams of hydroxyapatite/cubic centimeter. Source data are provided as a Source Data file.

Supplementary Figure 9

a

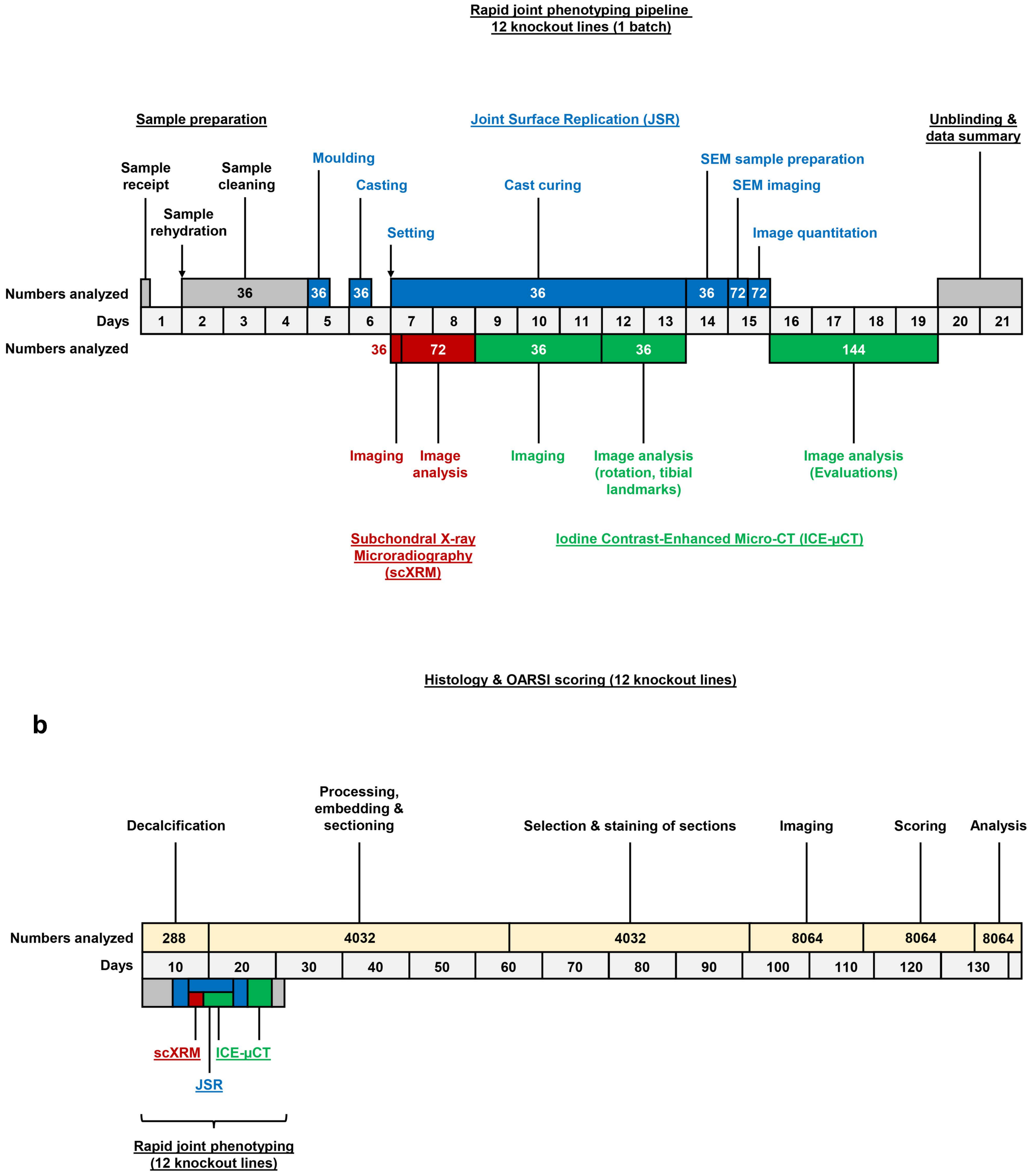

Origins of Bone and Cartilage Disease (OBCD) rapid-throughput joint phenotyping compared to Osteoarthritis Research Society International (OARSI) histological scoring

a. Timeline showing number of days and number of samples and images analysed for OBCD rapid-throughput joint phenotyping of 12 mutant mouse lines (n=3 mice required per line).

b. Timeline showing number of days and number of samples and histological sections analysed for phenotyping 12 mutant mouse lines by gold-standard OARSI scoring (n=12 mutant and 12 wild type mice required per line).

a

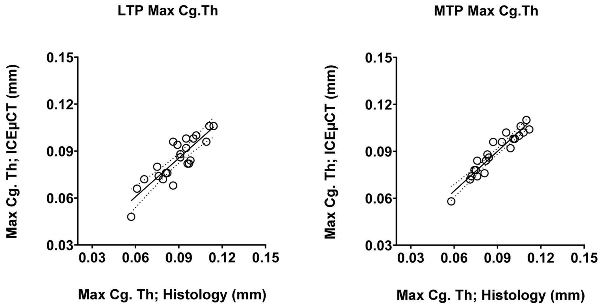

b

Linear regression	Medial Tibial Plateau (MTP)	Lateral Tibial Plateau (LTP)
Slope	0.847	0.824
R^2	0.905	0.755
P-value	<0.0001	<0.0001
Deviation from zero?	Significant (***)	Significant (***)
Equation	Y = 0.8469*X + 0.01380	Y = 0.8244*X + 0.01133

c

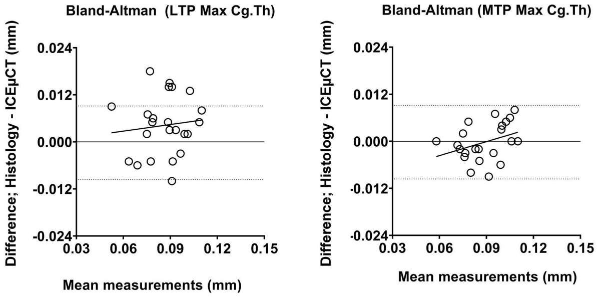

d

Bland-Altman plots		Medial Tibial Plateau (MTP)	Lateral Tibial Plateau (LTP)
Bias		-0.0002273 mm	0.004217 mm
SD of bias		0.00478 mm	0.007556 mm
95% limits of agreement	From	-0.009597 mm	-0.01059 mm
	To	0.009142 mm	0.01903 mm
Trendline			
R^2		0.125	0.011
Slope		0.119	0.056
P-value		0.106	0.630
Deviation from zero?		Not Significant	Not Significant
Equation		Y = 0.1190*X - 0.01080	Y = 0.05639*X - 0.0006577

e

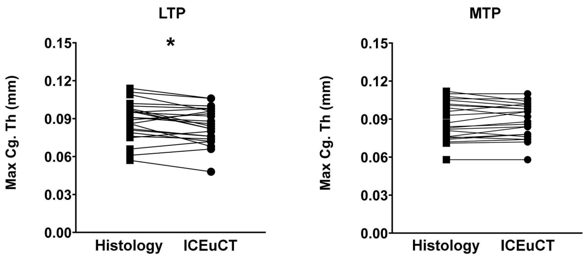

f

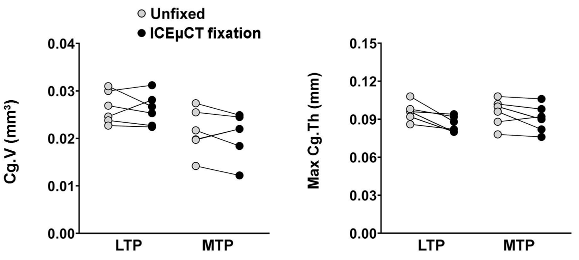

Validation of iodine contrast-enhanced µCT (ICEµCT)

a. Maximum cartilage thickness (max. Cg.Th, mm) determined by ICEµCT versus maximum cartilage thickness determined by histology (mm). Lateral tibial plateau (LTP); medial tibial plateau (MTP); linear regression and 95% confidence intervals.

b. Correlation (R^2) between cartilage thickness determined by ICEµCT and histology.

c. Bland-Altman plots comparing maximum cartilage thickness determined by ICEµCT versus histology. Linear regression and 95% limits of agreement are shown.

d. Table demonstrating that Bland-Altman analysis does not deviate from zero.

e. Max Cg.Th is significantly larger when measured by histology than when measured by ICEµCT, for LTP only (P=0.014, paired 2-tailed *t*-test assuming unequal variance, **P*<0.05).

f. Articular cartilage volume (Cg.V) and Max Cg.Th in samples under ICEµCT conditions (fixation in 10% neutral buffered formalin, stored in 70% ethanol, rehydrated 24-36h in phosphate buffered saline) are not significantly different from that of unfixed cartilage (Bonferroni-corrected *P*-value **P*<0.017). Cg.V (LTP): *P*=0.705, (MTP): *P*=0.500, Max. Cg.Th (LTP): *P*=0.0189, (MTP): *P*=0.135. mm; millimeter. Source data are provided as a Source Data file.

Supplementary Methods

Isolation of chondrocytes

For cohorts 1-2, the isolation of chondrocytes has been described previously[1] and the same protocol was followed for cohort 4. The protocol is described below. Osteochondral samples were transported in Dulbecco's modified Eagle's medium (DMEM)/F-12 (1:1) (Life Technologies) supplemented with 2mM glutamine (Life Technologies), 100U/mL penicillin, 100μg/mL streptomycin (Life Technologies), 2.5μg/mL amphotericin B (Sigma-Aldrich) and 50μg/mL ascorbic acid (Sigma-Aldrich) (serum-free media). Half of each sample was then taken forward for chondrocyte extraction. Cartilage was removed from the bone, dissected and washed twice in 1xPBS. Tissue was digested in 3mg/mL collagenase type I (Sigma-Aldrich) in serum-free media overnight at 37°C on a flatbed shaker. The resulting cell suspension was passed through a 70μm cell strainer (Fisher Scientific) and centrifuged at 400g for 10 minutes. Subsequently, the cell pellet was washed twice in serum-free media and centrifuged at 400g for 10 minutes. The resulting cell pellet was resuspended in serum-free media. Cells were counted using a haemocytometer and the viability checked using trypan blue exclusion (Invitrogen). The optimal cell number for spin column extraction from cells was between $4x10^6$ and $1x10^7$. Cells were then pelleted and homogenized.

For cohort 3, the extraction of chondrocytes has previously been described[2]. The protocol was based on that for cohorts 1, 2 and 4 above, and is highly similar, with differences summarized in the following. Each cartilage portion was minced with a scalpel and placed in 20mL of Dulbecco’s modified Eagle medium (Invitrogen) containing 10% foetal bovine serum (Invitrogen) and 6mg/mL collagenase A (Sigma).

The tissue culture flasks were incubated overnight to digest the cartilage pieces. The resulting cell suspension was passed through a 30µm filter (Miltenyi) and centrifuged at 400g for 10 minutes. The cell pellet was then re-suspended in 1mL of PBS and counted on a haemocytometer following 1:1 mixing with trypan blue to determine cell viability.

DNA, RNA and protein extraction

DNA, RNA, and protein extraction was carried out using Qiagen AllPrep DNA/RNA/Protein Mini Kit following manufacturer's instructions. For cohort 3, minor modifications were made to the approach as previously described[2] and the protocol is summarized below. Cells were lysed in 350µL RLT buffer containing 3.5µL β-mercaptoethanol and centrifuged in a Qiashredder column (Qiagen) for 2min at 20,800g. RNA, DNA and protein were purified using a series of column based centrifugation steps and RNA was eluted in 34µL RNase-free water, DNA in 100µL EB buffer and the protein pellet solubilized in 100µL 10% sodium dodecyl sulphate in PBS. DNA and RNA preparations were re-precipitated to remove buffer salts, and to concentrate the final product 20µL 3M sodium acetate, pH 5.5 (Ambion) was added per 100µL of DNA or RNA solution and mixed gently. Then, 283µL of molecular grade ethanol (Sigma) was added per 100µL of DNA or RNA solution to precipitate the nucleic acids and the mixture placed at -20°C overnight. Samples were centrifuged at 20,800g for 30 minutes and the supernatant removed, the pellet was washed with 900µL 70% ethanol and the nucleic acids re-pelleted by spinning at 20,800g for 10 minutes. Nucleic acids were resuspended in 32µL DNase/RNase free water. Samples were frozen at -80°C (cohorts 1, 2, 4) or -70°C (cohort 3) prior to assays.

RNA sequencing

We purified poly-A tailed RNA (mRNA) from total RNA using Illumina's TruSeq RNA Sample Prep v2 kits. We then fragmented the mRNA using metal ion-catalyzed hydrolysis and synthesized a random-primed cDNA library. The resulting double-strand cDNA was used as the input to a standard Illumina library prep, whereby ends were repaired to produce blunt ends by a combination of fill-in reactions and exonuclease activity. We performed A-tailing to allow samples to be pooled, by adding an “A” base to the blunt ends and ligation to Illumina Paired-end Sequencing adapters containing unique index sequences. Due to better performance, the 10-cycle PCR amplification of libraries was carried out using KAPA Hifi Polymerase. A post-PCR Agilent Bioanalyzer was used to quantify samples, followed by sample pooling and size-selection of pools using the LabChip XT Caliper. The multiplexed libraries were sequenced on the Illumina HiSeq 2000 for cohort 1 and HiSeq 4000 for cohorts 2-4 (75bp paired-ends). Sequenced data underwent initial analysis and quality control on reads as standard. This yielded gene expression data for samples from 110 patients.

Proteomics

Proteomics analysis was performed on cartilage samples from 103 patients. For cohort 1, all steps of protein digestion, 6-plex TMT labelling, peptide fractionation and LC-MS analysis on the Dionex Ultimate 3000 UHPLC system coupled with the high-resolution LTQ Orbitrap Velos mass spectrometer (Thermo Scientific), were previously described[1] and details are given below. The sample preparation protocol formed the basis of processing for cohorts 2-4 using 10-plex TMT labelling and an Orbitrap Fusion Tribrid Mass Spectrometer (Thermo Scientific) with otherwise only minor alterations, as described below.

Proteomics: Cohort 1

Protein digestion and TMT labeling

The protein content of each sample was precipitated by the addition of 30μL TCA 8M at 4°C for 30 min. The protein pellets were washed twice with ice cold acetone and finally re-suspended in 40μL 0.1M triethylammonium bicarbonate, 0.05% SDS with pulsed probe sonication. Protein concentration was measured with Quick Start Bradford Protein Assay (Bio-Rad) according to manufacturer's instructions. Aliquots containing 30μg of total protein were prepared for trypsin digestion. Cysteine disulfide bonds were reduced by the addition of 2μL 50mM tris-2-carboxymethyl phosphine (TCEP) followed by 1h incubation in a heating block at 60°C. Cysteine residues were blocked by the addition of 1μL 200mM freshly prepared Iodoacetamide (IAA) solution and 30min incubation at room temperature in the dark. Trypsin (Pierce, MS grade) solution was added to a final concentration 70ng/μL to each sample for overnight digestion. After proteolysis the peptide samples were diluted to 100μL with 0.1M TEAB buffer. A 41μL volume of anhydrous acetonitrile was added to each TMT 6-plex reagent (Thermo Scientific) vial and after vortex mixing the content of each TMT vial was transferred to each sample tube. Labeling reaction was quenched with 8μL 5% hydroxylamine for 15 min after 1h incubation at room temperature. Samples were pooled and the mixture was dried with SpeedVac concentrator and stored at -20°C until high-pH Reverse Phase (RP) fractionation.

Peptide fractionation

Offline peptide fractionation based on high pH Reverse Phase (RP) chromatography was performed using the Waters, XBridge C18 column (2.1 x 150mm, 3.5μm, 120Å) on a Dionex Ultimate 3000 HPLC system equipped with autosampler. Mobile phase (A) was composed of 0.1% ammonium hydroxide and mobile phase (B) was

composed of 100% acetonitrile, 0.1% ammonium hydroxide. The TMT labelled peptide mixture was reconstituted in 100μL mobile phase (A), centrifuged and injected for fractionation. The multi-step gradient elution method at 0.2mL/min was as follows: for 5 minutes isocratic at 5% (B), for 35min gradient to 35% (B), gradient to 80% (B) in 5min, isocratic for 5minutes and re-equilibration to 5% (B). Signal was recorded at 280nm and fractions were collected in a time-dependent manner every one minute. The collected fractions were dried with SpeedVac concentrator and stored at -20°C until LC-MS analysis.

LC-MS analysis

LC-MS analysis was performed on the Dionex Ultimate 3000 UHPLC system coupled with the high-resolution LTQ Orbitrap Velos mass spectrometer (Thermo Scientific). Each peptide fraction was reconstituted in 40μL 0.1% formic acid and a volume of 10μL was loaded to the Acclaim PepMap 100, 100μm × 2cm C18, 5μm, 100Ȧ trapping column with a user modified injection method at 10μL/min flow rate. The sample was then subjected to a multi-step gradient elution on the Acclaim PepMap RSLC (75μm × 50cm, 2μm, 100Å) C18 capillary column (Dionex) retrofitted to an electrospray emitter (New Objective, FS360-20-10-N-20-C12) at 45°C. Mobile phase (A) was composed of 96% H_2O, 4% DMSO, 0.1% formic acid and mobile phase (B) was composed of 80% acetonitrile, 16% H_2O, 4% DMSO, 0.1% formic acid. The gradient separation method at flow rate 300nL/min was as follows: for 95min gradient to 45% B, for 5min up to 95% B, for 8min isocratic at 95% B, re-equilibration to 5% B in 2min, for 10min isocratic at 5% B.

The ten most abundant multiply charged precursors within 380-1500m/z were selected with FT mass resolution of 30,000 and isolated for HCD fragmentation with isolation

width 1.2Th. Normalized collision energy was set at 40 and the activation time was 0.1ms for one microscan. Tandem mass spectra were acquired with FT resolution of 7,500 and targeted precursors were dynamically excluded for further isolation and activation for 40 seconds with 10ppm mass tolerance. FT max ion time for full MS experiments was set at 200ms and FT MSn max ion time was set at 100ms. The AGC target vales were 3×10^6 for full FTMS and 1×10^5 for MSn FTMS. The DMSO signal at m/z 401.922718 was used as a lock mass.

Proteomics: Cohorts 2-4

Protein digestion and TMT labeling

The protein content of each sample was precipitated by the addition of 30µL TCA 8M at 4°C for 30min. The protein pellets were washed twice with ice cold acetone and finally re-suspended in 40µL 0.1M triethylammonium bicarbonate, 0.1% SDS with pulsed probe sonication. Equal aliquots containing at least 10µg of total protein were reduced with 5mM TCEP for 1h at 60°C, alkylated with 10mM Iodoacetamide and subjected to overnight trypsin (70ng/µL) digestion. TMT 10-plex (Thermo Scientific) labelling was performed according to manufacturer's instructions at equal amounts of tryptic digests. Samples were pooled and the mixture was dried with SpeedVac concentrator and stored at -20°C until peptide fractionation.

Peptide fractionation

Offline peptide fractionation was based on high pH Reverse Phase (RP) chromatography using the Waters, XBridge C18 column (2.1 x 150mm, 3.5µm) on a Dionex Ultimate 3000 HPLC system. Mobile phase A was 0.1% ammonium hydroxide and mobile phase B 100% acetonitrile, 0.1% ammonium hydroxide. The TMT labelled

peptide mixture was dissolved in 100μL mobile phase A, centrifuged and injected for fractionation. The gradient elution method at 0.2mL/min included the following steps: 5 minutes isocratic at 5% B, for 35 min gradient to 35% B, gradient to 80% B in 5 min, isocratic for 5 minutes and re-equilibration to 5% B. Signal was recorded at 280nm and fractions were collected every one minute. For cohort 4, peptide fractionation was performed on reversed-phase OASIS HLB cartridges at high pH and up to 9 fractions (10-25% acetonitrile elution steps) were collected for each set. The collected fractions were dried with SpeedVac concentrator and stored at -20°C until LC-MS analysis.

LC-MS analysis

LC-MS analysis was performed on the Dionex Ultimate 3000 UHPLC system coupled with the Orbitrap Fusion Tribrid Mass Spectrometer (Thermo Scientific). Each peptide fraction was reconstituted in 40μL 0.1% formic acid and a volume of 7μL was loaded to the Acclaim PepMap 100, 100μm × 2cm C18, 5μm, 100Å trapping column with the μLPickUp mode at 10μL/min flow rate. The sample was then analysed with a gradient elution on the Acclaim PepMap RSLC (75μm × 50cm, 2μm, 100Å) C18 capillary column retrofitted to an electrospray emitter (New Objective, FS360-20-10-D-20) at 45°C. Mobile phase A was 0.1% formic acid and mobile phase B was 80% acetonitrile, 0.1% formic acid. The gradient method at flow rate 300nL/min was: for 90min gradient to 38% B, for 5min up to 95% B, for 13min isocratic at 95% B, re-equilibration to 5% B in 2min, for 10min isocratic at 10% B. Precursors were selected with 120k mass resolution, AGC 3×10^5 and IT 100ms in the top speed mode within 3sec and were targeted for CID fragmentation with quadrupole isolation width 1.2Th. Collision energy was set at 35% with AGC 1×10^4 and IT 35ms. MS3 quantification spectra were acquired with further HCD fragmentation of the top 10 most abundant CID fragments

isolated with Synchronous Precursor Selection (SPS) excluding neutral losses of maximum m/z 18. Iontrap isolation width was set at 0.7Th for MS1 isolation, collision energy was applied at 55% and the AGC setting was at 6×10^4 with 100ms IT. The HCD MS3 spectra were acquired within 110-400m/z with 60k resolution. Targeted precursors were dynamically excluded for further isolation and activation for 45 seconds with 7ppm mass tolerance. Cohort 4 was analyzed at the MS2 level with a top15 HCD method (CE 40%, 50k resolution) and a maximum precursor intensity threshold of 5×10^7 using the same MS1 parameters as above in a 360min gradient.

Quantification of RNA levels

We used samtools v1.3.1[3] and biobambam v0.0.191[4] to convert cram to fasq files after exclusion of reads that failed QC. We applied FastQC v0.11.5 to check sample quality[5] and excluded 7 samples.

We obtained transcript-level quantification using salmon 0.8.2[6] (with --gcBias and --seqBias flags to account for potential biases) and the GRCh38 cDNA assembly release 87 downloaded from Ensembl [http://ftp.ensembl.org/pub/release-87/fasta/homo_sapiens/cdna/]. We used tximport[7] to convert transcript-level to gene-level scaled transcripts per million (TPM) estimates, with estimates for 39,037 genes based on Ensembl gene IDs.

We excluded 4 samples due to low mapping rate (<80%), 8 samples due to non-European ancestry, 15 samples due to low RIN (<5), 3 samples due to abnormal gene read density plots.

The final gene expression dataset included 87 patients' low-grade and 95 high-grade cartilage samples with 15,249 genes that showed counts per million (CPM) of ≥1 in ≥40 samples (matched low-grade and high-grade samples from 83 patients).

Quantification of protein levels

To carry out protein identification and quantification, we submitted the mass spectra to SequestHT search in Proteome Discoverer 2.1. The precursor mass tolerance was set at 30ppm (Orbitrap Velos data, cohort 1) or 20ppm (Fusion data, cohorts 2-4). For the CID spectra, we set the fragment ion mass tolerance to 0.5Da; for the HCD spectra, to 0.02Da. Spectra were searched for fully tryptic peptides with maximum 2 miss-cleavages and minimum length of 6 amino acids. We specified static modifications as TMT-6-plex at N-terminus, K and Carbamidomethyl at C; dynamic modifications included deamidation of N,Q and oxidation of M. For each peptide, we allowed for a maximum two different dynamic modifications with a maximum of two repetitions. We used the Percolator node to estimate peptide confidence. We set the peptide false discovery rate (FDR) at 1% and based validation on the q-value and decoy database search. We searched all spectra against a UniProt fasta file that contained 20,165 reviewed human entries. The Reporter Ion Quantifier node included a TMT-6-plex (Velos data, cohort 1) or TMT-10-plex (Fusion data, cohorts 2-4) custom Quantification Method with integration window tolerance at 20ppm or 15ppm, respectively. As integration methods, we used the Most Confident Centroid at the MS2 or MS3 level. We only used peptides uniquely belonging to protein groups for quantification.

We excluded samples from 4 patients due to non-European ancestry. The final dataset included low-grade and high-grade cartilage samples each from 99 patients, with 4,801 proteins observed in ≥30% of samples, and 1,677 proteins in all samples, in line with the resolution depth of the isobaric labelling method employed. To account for protein loading, abundance values were normalized by the sum of all protein abundances in a given sample, then log2-transformed and quantile normalized.

Differential RNA expression between high-grade and low-grade cartilage

We tested differential expression of 15,249 genes between high-grade and low-grade cartilage using paired samples from 83 patients. To detect robust gene expression differences, we carried out analyses using different software packages as recommended in a landmark survey of best practices[8], applying limma[9], edgeR[10], and DESeq2[11]. We also tested 5 analysis designs with different options to account for technical variation, including SVAseq[12]. In particular, we tested for differential expression using:

1) A paired analysis of intact and degraded samples (i.e. specifying patient ID as covariate)
2) A paired analysis of intact and degraded samples, with 10 additional covariates accounting for technical variation identified by SVAseq[12]
3) A paired analysis of intact and degraded samples, with 10 RNA sequencing batches as covariates
4) An unpaired analysis of intact and degraded samples
5) An unpaired analysis of intact and degraded samples, with 19 additional covariates accounting for technical variation identified by SVAseq

We tested for differential expression using the following R packages:

I. Limma[9] (with lmFit and eBayes), after applying limma-voom[13] to remove heteroscedasticity

II. DESeq2[11], separately with and without outlier filtering/replacement (minReplicatesForReplace=Inf, cooksCutoff=FALSE options)

III. EdgeR[10], using the likelihood ratio test (glmFit and glmLRT functions), and separately, using the F test (glmQLFit and glmQLFTest functions)

Here and elsewhere, we used Ensembl38p10 to identify genes with uniquely corresponding Ensembl gene ID and gene name (13,737 of 15,249 genes in the RNA data).

In each analysis design and method, we used a 5% False Discovery Rate (FDR) threshold to correct for multiple testing. As the final step, we applied a conservative approach and considered a gene “significantly differentially expressed” between low-grade and high-grade cartilage if it showed significant differential expression across all analysis designs and testing methods (2,557 genes, including 2,418 with uniquely corresponding Ensembl gene ID and gene name).

Differential protein abundance between high-grade and low-grade cartilage

We performed differential analysis for 4,801 proteins that were measured in ≥30% of patients, applying limma[9] to paired samples from 99 patients. Significance was defined at 5% FDR to correct for multiple testing, yielding 2,233 proteins with significant differential abundance (2,019 proteins with uniquely corresponding Ensembl gene ID and gene name).

As batch effects in proteomics data can be pervasive[14,15], paired samples from any patient were always assayed in the same 6-plex (cohort 1) or 10-plex (cohorts 2-4).

Supplementary References

1. Steinberg, J.*, et al.* Integrative epigenomics, transcriptomics and proteomics of patient chondrocytes reveal genes and pathways involved in osteoarthritis. *Sci. Rep.* **7**, 8935 (2017).

2. Steinberg, J.*, et al.* Widespread epigenomic, transcriptomic and proteomic differences between hip osteophytic and articular chondrocytes in osteoarthritis. *Rheumatology (Oxford)* **57**, 1481-1489 (2018).

3. Li, H.*, et al.* The Sequence Alignment/Map format and SAMtools. *Bioinformatics* **25**, 2078-2079 (2009).

4. Tischler, G. & Leonard, S. biobambam: tools for read pair collation based algorithms on BAM files. *Source Code Biol. Med.* **9**, 13-13 (2014).

5. Andrews, S. FastQC: a quality control tool for high throughput sequence data. Available online at: http://www.bioinformatics.babraham.ac.uk/projects/fastqc. (2010).

6. Patro, R., Duggal, G., Love, M.I., Irizarry, R.A. & Kingsford, C. salmon provides fast and bias-aware quantification of transcript expression. *Nat. Meth.* **14**, 417-419 (2017).

7. Soneson, C., Love, M. & Robinson, M. Differential analyses for RNA-seq: transcript-level estimates improve gene-level inferences [version 1; referees: 2 approved]. *F1000Research* **4**, 1521 (2015).

8. Conesa, A.*, et al.* A survey of best practices for RNA-seq data analysis. *Genome Biol.* **17**, 13 (2016).

9. Ritchie, M.E.*, et al.* limma powers differential expression analyses for RNA-sequencing and microarray studies. *Nucleic Acids Res.* **43**, e47 (2015).

10. McCarthy, D.J., Chen, Y. & Smyth, G.K. Differential expression analysis of multifactor RNA-Seq experiments with respect to biological variation. *Nucleic Acids Res.* **40**, 4288-4297 (2012).

11. Love, M.I., Huber, W. & Anders, S. Moderated estimation of fold change and dispersion for RNA-seq data with DESeq2. *Genome Biol.* **15**, 550 (2014).

12. Leek, J.T. svaseq: removing batch effects and other unwanted noise from sequencing data. *Nucleic Acids Res.* **42**, e161 (2014).

13. Law, C.W., Chen, Y., Shi, W. & Smyth, G.K. voom: precision weights unlock linear model analysis tools for RNA-seq read counts. *Genome Biol.* **15**, R29 (2014).

14. Gregori, J.*, et al.* Batch effects correction improves the sensitivity of significance tests in spectral counting-based comparative discovery proteomics. *J. Proteom.* **75**, 3938-3951 (2012).

15. Kuligowski, J.*, et al.* Detection of batch effects in liquid chromatography-mass spectrometry metabolomic data using guided principal component analysis. *Talanta* **130**, 442-448 (2014).
