# Supplementary material for: Accelerating functional gene discovery in osteoarthritis
Source: Nat Commun. 2021 Jan 20;12:467. doi: 10.1038/s41467-020-20761-5 (PMC7817695; doi:10.1038/s41467-020-20761-5)
Supplement: Supplementary file 15 — Supplementary Data 14 [file 41467_2020_20761_MOESM15_ESM.docx]

**Supplementary Data 14**

**ImageJ macros for automated quantitation of joint surface replication (JSR) and subchondral X-ray microradiography (scXRM)**

*All macros were generated using ImageJ1.44. Text between square brackets is descriptive and does not form part of the macro.*

**JSR Macros**

***Macro 1***

*[****Description:*** *This macro clears outside freehand selection of plateaux in all open images, saves as .tiff to selectable destination directory, appends 'plateau' to end of file name and closes images.]*

dir2 = getDirectory("Choose Destination Directory ");

setBatchMode(true);

imgArray = newArray(nImages);

for (i=0; i<nImages; i++) {

selectImage(i+1);

imgArray[i] = getTitle();

}

for (i=0; i< imgArray.length; i++) {

selectImage(imgArray[i]);

run("Clear Outside");

saveAs("TIFF", dir2 +imgArray[i]+"_plateau");

close();

}

setBatchMode(false);

***Macro 2***

*[****Description:*** *This macro removes bright outliers 4 pixels and below from all images in selectable source directory, saves as .tiff to selectable destination directory, appends '_RO ' to the end of the file name and closes images.]*

dir1 = getDirectory("Choose Source Directory ");

dir2 = getDirectory("Choose Destination Directory ");

list = getFileList(dir1);

setBatchMode(true);

for (i=0; i<list.length; i++) {

showProgress(i+1, list.length);

open(dir1+list[i]);

run("Remove Outliers...", "radius=4 threshold=50 which=Bright");

saveAs("TIFF", dir2+list[i]+"_RO");

close();

}

***Macro 3***

[**Description:** *This macro finds edges on images of plateaux in selectable source directory, saves as .tiff to selectable destination directory and appends ‘edges’ to the file name.]*

dir1 = getDirectory("Choose Source Directory ");

dir2 = getDirectory("Choose Destination Directory ");

list = getFileList(dir1);

setBatchMode(true);

for (i=0; i<list.length; i++) {

showProgress(i+1, list.length);

open(dir1+list[i]);

run("Find Edges");

saveAs("TIFF", dir2+list[i]+"edges");

close();

}

***Macro 4***

[**Description:** *This macro saves all open files into selectable destination directory, appends 'cleaned' to the title name, and closes images.]*

dir2 = getDirectory("Choose Destination Directory ");

setBatchMode(true);

imgArray = newArray(nImages);

for (i=0; i<nImages; i++) {

selectImage(i+1);

imgArray[i] = getTitle();

}

for (i=0; i< imgArray.length; i++) {

selectImage(imgArray[i]);

saveAs("TIFF", dir2 +imgArray[i]+"_cleaned");

close();

}

setBatchMode(false);

***Macro 5***

*[****Description:*** *This macro saves all open images as .tiff to selectable destination directory, appends ‘Thresh ' to file name and closes images.]*

dir2 = getDirectory("Choose Destination Directory ");

setBatchMode(true);

imgArray = newArray(nImages);

for (i=0; i<nImages; i++) {

selectImage(i+1);

imgArray[i] = getTitle();

}

for (i=0; i< imgArray.length; i++) {

selectImage(imgArray[i]);

saveAs("TIFF", dir2 +imgArray[i]+"_Thresh");

close();

}

setBatchMode(false);

***Macro 6***

*[****Description:*** *This macro analyses all images in selectable source folder, selects all particles with size 20-infinity and circularity 0-0.5 and saves outlines as new image.tiff in selectable destination directory.]*

dir1 = getDirectory("Choose Source Directory ");

dir2 = getDirectory("Choose Destination Directory ");

list = getFileList(dir1);

setBatchMode(true);

for (i=0; i<list.length; i++) {

showProgress(i+1, list.length);

open(dir1+list[i]);

//run("Threshold...");

setAutoThreshold("Minimum dark");

setThreshold(253, 255);

run("Convert to Mask");

run("Analyze Particles...", "size=20-Infinity circularity=0.00-0.5 show=[Bare Outlines] display summarize");

saveAs("Tiff", dir2+list[i]+"_bare outlines");

close();

}

**scXRM Macros**

***Macro 7***

*[****Description:*** *This macro stretches pixel information between the pixel value for the lowest-density standard (plastic) and the highest density standard (steel), applies a 16-colour look-up table and saves as a new image.]*

Dialog.create("Set Standards");

Dialog.addNumber("Steel mode grey level", 779, 0, 5, "");

Dialog.addNumber("Plastic mode grey level", 8769, 0, 5, "");

Dialog.addNumber("Minimum grey level", 0, 0, 5, "");

Dialog.addNumber("Maximum grey level", 12031, 0, 5, "");

Dialog.show;

steel = Dialog.getNumber;

plastic = Dialog.getNumber;

low = Dialog.getNumber;

high = Dialog.getNumber;

strl = steel

strh = plastic

min=low+(high-plastic)

max=high-(steel-low)

setBatchMode(true);

setMinAndMax(min,max);

run("Invert");

original = getTitle();

width = getWidth();

height = getHeight();

setBatchMode(true);

pix = newArray(width*height);

for (y=0; y<height; y++){

for (x=0; x<width; x++){

pix[y*width+x] = (getPixel(x,y)-min)*255/(max-min);

}

}

close();

newImage(original+"_normalised"+" "+strl+"_"+strh+" 16colour.tif", "8-bit black", width, height, 1);

selectWindow(original+"_normalised"+" "+strl+"_"+strh+" 16colour.tif");

for (y=0; y<height; y++){

for (x=0; x<width; x++){

setPixel(x,y, pix[y*width+x]);

}

}

run("16_colors");

setBatchMode(false);
