# Supplementary material for: Accelerating functional gene discovery in osteoarthritis
Source: Nat Commun. 2021 Jan 20;12:467. doi: 10.1038/s41467-020-20761-5 (PMC7817695; doi:10.1038/s41467-020-20761-5)
Supplement: Supplementary file 20 — Reporting Summary [file 41467_2020_20761_MOESM20_ESM.pdf]

nature research

Corresponding author(s): Graham R Williams & JH Duncan Bassett

Last updated by author(s): Nov 26, 2020

Reporting Summary

Nature Research wishes to improve the reproducibility of the work that we publish. This form provides structure for consistency and transparency in reporting. For further information on Nature Research policies, see Authors & Referees and the Editorial Policy Checklist .

Statistics

For all statistical analyses, confirm that the following items are present in the figure legend, table legend, main text, or Methods section.

n/a	Confirmed	
☐	☒	The exact sample size (*n*) for each experimental group/condition, given as a discrete number and unit of measurement
☐	☒	A statement on whether measurements were taken from distinct samples or whether the same sample was measured repeatedly
☐	☒	The statistical test(s) used AND whether they are one- or two-sided<br>*Only common tests should be described solely by name; describe more complex techniques in the Methods section.*
☐	☒	A description of all covariates tested
☐	☒	A description of any assumptions or corrections, such as tests of normality and adjustment for multiple comparisons
☐	☒	A full description of the statistical parameters including central tendency (e.g. means) or other basic estimates (e.g. regression coefficient) AND variation (e.g. standard deviation) or associated estimates of uncertainty (e.g. confidence intervals)
☐	☒	For null hypothesis testing, the test statistic (e.g. *F*, *t*, *r*) with confidence intervals, effect sizes, degrees of freedom and *P* value noted<br>*Give P values as exact values whenever suitable.*
☒	☐	For Bayesian analysis, information on the choice of priors and Markov chain Monte Carlo settings
☒	☐	For hierarchical and complex designs, identification of the appropriate level for tests and full reporting of outcomes
☒	☐	Estimates of effect sizes (e.g. Cohen's *d*, Pearson's *r*), indicating how they were calculated

Our web collection on statistics for biologists contains articles on many of the points above.

Software and code

Policy information about availability of computer code

Data collection	ImageJ1.44 (RRID:SCR_003070)<br>Scanco µCT Tomography software v6.4-2/Open VMS<br>Xming 6.9.0.31 (Colin Harrison; http://www.straightrunning.com/XmingNotes/)<br>PuTTY 0.62 (Simon Tatham; https://www.putty.org)<br>ImageSnapper v1.0 (Tescan, UK; www.tescan.co.uk)<br>FastQC 0.11.5 (Simon Andrews; http://www.bioinformatics.babraham.ac.uk/projects/fastqc)<br>Salmon 0.8.2 (Patro et al., 2017; https://github.com/COMBINE-lab/salmon)<br>Proteome Discoverer 2.1 software (Thermo Fisher Scientific, Waltham, MA, USA; Cat# OPTON-30812)
Data analysis	Microsoft Office 2016<br>Prism GraphPad 8 (GraphPad Software Inc; RRID:SCR_002798)<br>R Project for Statistical Computing (http://www.r-project.org; RRID:SCR_001905)<br>R packages - tximport (https://bioconductor.org/packages/release/bioc/html/tximport.html), limma (https://bioconductor.org/packages/release/bioc/html/limma.html), DESeq2 (https://bioconductor.org/packages/release/bioc/html/DESeq2.html), edgeR (https://bioconductor.org/packages/release/bioc/html/edgeR.html)<br>SPSS v26 (IBM SPSS Statistics RRID:SCR_002865)<br><br>Code provided in Supplementary Data 14 has been shared on the public repository Github accessible at https://github.com/Molendo/OBCD. Repository: Molendo. Project: OBCD (search term: Molendo/OBCD).

For manuscripts utilizing custom algorithms or software that are central to the research but not yet described in published literature, software must be made available to editors/reviewers. We strongly encourage code deposition in a community repository (e.g. GitHub). See the Nature Research guidelines for submitting code & software for further information.

Data

Policy information about availability of data

All manuscripts must include a data availability statement. This statement should provide the following information, where applicable:

- Accession codes, unique identifiers, or web links for publicly available datasets
- A list of figures that have associated raw data
- A description of any restrictions on data availability

Data Availability Statement
Source data are provided with this paper. All datasets generated and/or analyzed during the current study are included in Supplementary and Source Data, and available from the corresponding authors on reasonable request or as detailed below. Figures 1, 3-7, and Supplementary Figures 1-8 and 10 have associated raw data (included in the Supplementary Data and Source Data).

Additionally, all raw RNA sequencing data have been deposited in the European Genome-Phenome Archive [EGA; https://www.ebi.ac.uk/ega/home) with the identifiers EGAS00001002255, EGAD00001003355 (n=17), EGAD00001003354 (n=9), EGAD00001001331 (n=12). Proteomics data have been deposited in the PRoteomics IDEntifications database (PRIDE; https://www.ebi.ac.uk/pride/archive/) with the identifiers PXD006673, PXD00202014 and PDX014666, username: reviewer90654@ebi.ac.uk, password: AoeRBm3e).

Public databases used in this study are: BioGPS (http://biogps.org/#goto=welcome), Deciphering the Mechanisms of Developmental Disorders database (DMDD; https://dmdd.org.uk/), European Mutant Mouse Archive (EMMA; https://www.infrafrontier.eu/), European Bioinformatics Institute Genome-wide Association Studies database (EBI GWAS Catalogue, https://www.ebi.ac.uk/gwas/), Google Scholar (https://scholar.google.co.uk/), International Mouse Phenotyping Resource of Standardized Screens (IMPReSS; https://www.mousephenotype.org/impress), Mouse Genome Informatics database (http://www.informatics.jax.org/), Online Mendelian Inheritance in Man (OMIM, https://www.omim.org/), PubMed (https://www.ncbi.nlm.nih.gov/pubmed/) and SkeletalVis28 (http://phenome.manchester.ac.uk/).

There are no restrictions on data availability.

Field-specific reporting

Please select the one below that is the best fit for your research. If you are not sure, read the appropriate sections before making your selection.

☒ Life sciences ☐ Behavioural & social sciences ☐ Ecological, evolutionary & environmental sciences

For a reference copy of the document with all sections, see nature.com/documents/nr-reporting-summary-flat.pdf

Life sciences study design

All studies must disclose on these points even when the disclosure is negative.

Sample size

Sample sizes were based on power calculations performed using the coefficient of variance (for parameters that were normally-distributed in a wild-type population of 100 animals) or the percentage median absolute deviation from the median (median absolute deviation from the median (MAD)/median) for non-normally distributed parameters.

Data exclusions

For analysis of 50 unselected mouse knockout lines generated by the International Mouse Phenotyping Consortium (IMPC), no samples were excluded.

For analysis of 100 wild-type samples, 13 samples were excluded based on pre-determined criteria including iatrogenic damage to the joint that occurred during sample preparation.

For histological analysis of mice undergoing surgical provocation of osteoarthritis, one mouse was excluded due to unsuccessful surgery. 4 mice were excluded due to surgery damaging ligaments in the knee that were not the target ligaments of the surgery. These exclusion criteria were pre-determined based on published accounts of the surgical method and included detection of subchondral bone erosion and calcification of the cruciate ligaments (Glasson, Osteoarthritis and Cartilage, 2007:15, 1061e1069).

Replication

A repeatability study was performed for the new methods described. To determine the repeatability of each method, 7 wild-type and 6 mutant samples were selected that covered the full spectrum of phenotype severity. Samples were blinded and analyzed five times in a random order, and in a different random order for each method. Mean, standard error of the mean, number of standard deviations from baseline mean, absolute precision error (standard deviation; PE(SD), and precision error as percentage of coefficient of variation (PE(%CV) and two-way mixed-model intraclass correlation coefficients (absolute agreement) with 95% confidence intervals were calculated for repeated analyses by a single rater.

Randomization

For analysis of 50 unselected mouse knockout strains generated by the IMPC, 7 samples were available for each genotype. In cases where fewer than 7 samples were analysed, samples were randomly selected for analysis.

To generate the wild-type reference range, 100 wild-type samples were randomly selected for analysis.

For surgical provocation of osteoarthritis in wild-type male mice, 2 mice per cage of 4 were randomly allocated for analysis by either rapid

	joint phenotyping or histology.
Blinding	All mouse samples were analysed in blinded batches for all data collection and analyses. All human samples came from osteoarthritis patients, so no blinding was performed to disease status. RNA and proteomics measurements were quantitative and performed automatically using the same procedure for all patients. During the statistical analysis, cartilage tissue degradation was used as the variable of interest, so no blinding was possible.

Reporting for specific materials, systems and methods

We require information from authors about some types of materials, experimental systems and methods used in many studies. Here, indicate whether each material, system or method listed is relevant to your study. If you are not sure if a list item applies to your research, read the appropriate section before selecting a response.

Materials & experimental systems

n/a	Involved in the study
☒	☐ Antibodies
☒	☐ Eukaryotic cell lines
☒	☐ Palaeontology
☐	☒ Animals and other organisms
☐	☒ Human research participants
☒	☐ Clinical data

Methods

n/a	Involved in the study
☒	☐ ChIP-seq
☒	☐ Flow cytometry
☒	☐ MRI-based neuroimaging

Animals and other organisms

Policy information about studies involving animals; ARRIVE guidelines recommended for reporting animal research

Laboratory animals	See Supplementary Data 12 for a complete list of animal lines used in this study. All details required to comply with ARRIVE guidelines are provided in the Experimental Models subsection of the Methods section.
Wild animals	No wild animals were used in the study.
Field-collected samples	No field collected samples were used in the study.
Ethics oversight	Animal experiments were approved by the Sanger or Imperial College Hammersmith Campus Animal Welfare Ethical Review Bodies (AWERB) as appropriate. Studies performed at the University of Chicago were approved by the Institutional Animal Care and Use Committee (IACUC) at Rush University Medical Center (16-077 and 15-033).

Note that full information on the approval of the study protocol must also be provided in the manuscript.

Human research participants

Policy information about studies involving human research participants

Population characteristics	We collected tissue samples from 115 patients undergoing total joint replacement surgery for osteoarthritis: 12 knee osteoarthritis patients (cohort 1; 2 women, 10 men, age 50-88 years, mean 68 years); 20 knee osteoarthritis patients (cohort 2; 14 women, 6 men, age 54-82 years, mean 70 years); 13 hip osteoarthritis patients (cohort 3; 8 women, 5 men, age 44-84 years, mean 62 years); 70 knee osteoarthritis patients (cohort 4; 42 women, 28 men, age 38-84 years, mean 70 years).
Recruitment	Not applicable
Ethics oversight	The study design and conduct complied with all relevant regulations regarding the use of human study participants and were conducted in accordance to the criteria set by the Declaration of Helsinki. All patients provided written, informed consent prior to participation.<br><br>This work was approved by Oxford NHS REC C (10/H0606/20 and 15/SC/0132). Samples from knee osteoarthritis patients were collected under Human Tissue Authority license 12182, Sheffield Musculoskeletal Biobank, University of Sheffield, UK; samples from hip osteoarthritis patients were collected under National Research Ethics approval reference 11/EE/0011, Cambridge Biomedical Research Centre Human Research Tissue Bank, Cambridge University Hospitals, UK.<br><br>This information has also been provided in the manuscript (Methods: Human sample collection)

Note that full information on the approval of the study protocol must also be provided in the manuscript.
