# Supplementary material for: Accelerating functional gene discovery in osteoarthritis
Source: Nat Commun. 2021 Jan 20;12:467. doi: 10.1038/s41467-020-20761-5 (PMC7817695; doi:10.1038/s41467-020-20761-5)
Supplement: Supplementary file 21 — Supplementary Software [file 41467_2020_20761_MOESM21_ESM.zip › Butterfield et al CODE AND SOFTWARE/Butterfield_Code and software submission.docx]

**Code and software submission**

We have written macros for the program ImageJ to allow for automation of image analysis. The macro code is provided in Supplementary Data 14. ImageJ can be downloaded free of charge at <https://imagej.nih.gov/ij/download.html>

1. System requirements:
   1. Software dependencies: ImageJ1.44 or above
   2. Tested on: ImageJ1.44 and 1.46
   3. Non-standard hardware required: none
2. Installation guide
   1. To install and run macros, follow instructions at <https://imagej.nih.gov/ij/docs/index.html>
   2. Order of macro usage and macro function description is in the Methods section of the manuscript, Supplementary Data 14, and below in Appendix
   3. Typical install time of ImageJ macros on a ‘normal’ desktop computer is is negligible
3. Demo
   1. Instructions to run macros on data are provided in the Methods section of the manuscript, Supplementary Data 14, and below in Appendix
   2. Expected output is described below
   3. Expected run time for each macro is negligible (<1min for large batches of approx. 50 samples)
4. Instructions for use
   1. Instructions to run macros on data are provided in the Methods section of the manuscript, Supplementary Data 14, and below in Appendix 1 and 2

## Appendix 1: Demo instructions for macros 1-6

1. Open Demo_Data_Butterfield_macros1-6.tif in ImageJ1.44
2. Freeform select tool to manually select plateau edge
3. To clear outside the plateau selection: install & run ***Macro 1*** (specify Destination folder)
4. To remove outliers 4 pixels in size and below: install & run ***Macro 2*** (specify Source and Destination folder)


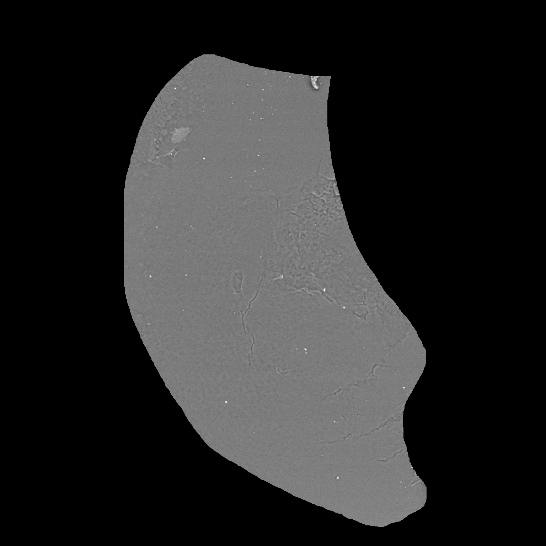

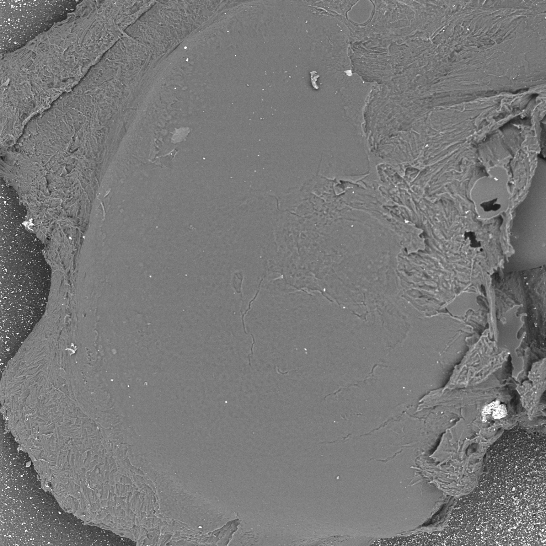

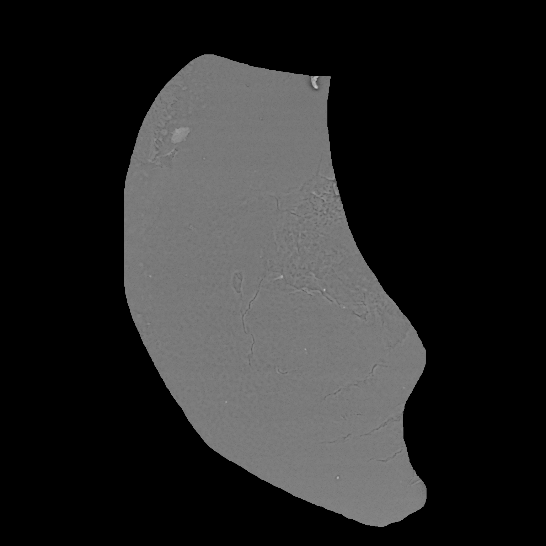


Cleared outside selection (plateaux)

Removed outliers

Original image

1. To get area of whole plateau:
   1. Open destination file from (4)
   2. Threshold manually by: Image>>Adjust>>Threshold. (set to minimum). Move top slider until whole plateau area is red >> Apply


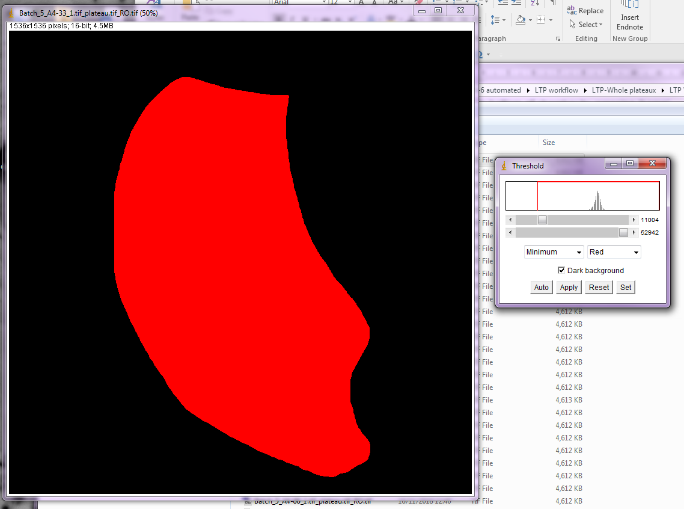


Minimum threshold

1. Repeat for each image in the batch and leave open and unsaved.
2. Batch save: install & run **Macro 5** (specify Destination folder)
3. Analyse area of each whole plateau: Install & run **Macro 6** (specify Source and Destination folder). Results will appear in a separate ‘results’ window
4. Copy the contents of the ‘summary’ window to Excel


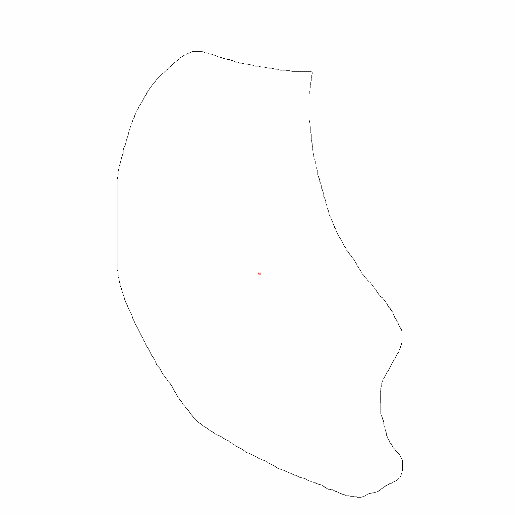


Results and whole plateau mask


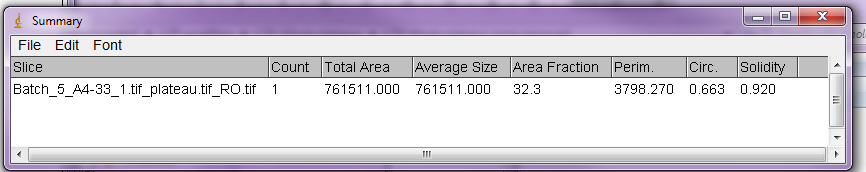


### To get area of damage:

- 1. Detect damage: Install & run ***Macro 3*** (ensure whole plateau is selected, and specify Source and Destination folder).
  2. To manually erase debris and artefacts, open all destination files from (7)
  3. Manually erase bubbles and soft tissue on plateau *(set foreground to black [Edit>>Options>>Colours] and brush size to ~50pix; right click on brush tool)*
  4. Repeat on all open files but **do not save**
  5. Batch save: install & run **Macro 4** (specify Destination folder)


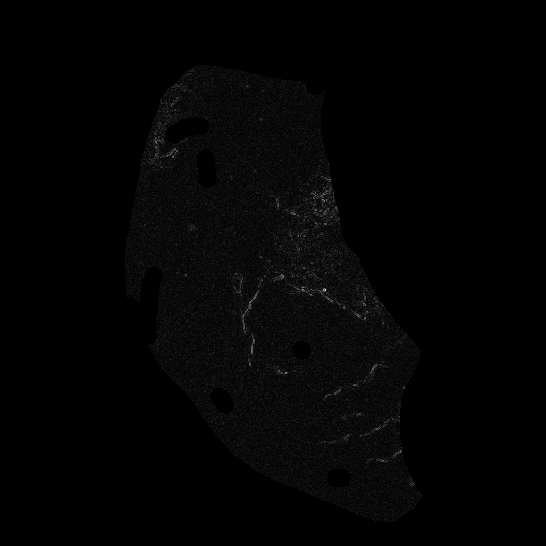


Manual clean


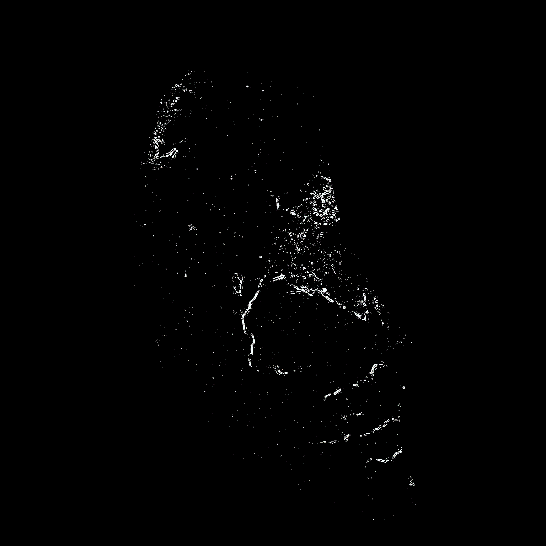


Thresholded


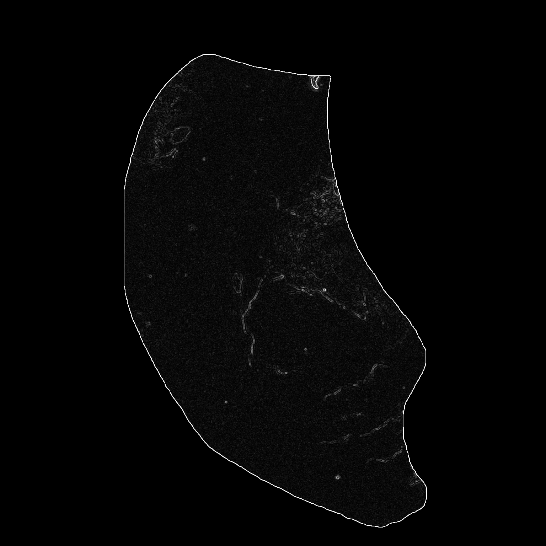


Find edges

### To threshold damage:

1. Open all destination images from (10e)
   1. Image>>Adjust>>Threshold (default) >> Apply to select the edges that most closely match the damage on the plateau.
   2. **Batch save:** install & run ***Macro 5*** (specify Destination folder)
2. To analyse damage area (Analyse Particles tool): Install & run the macro ***Macro 6*** (specify Source and Destination folder) IMPORTANT: Compare each mask with the original image and if it is not a true representation of the damage on the plateau, delete the summary line in the ‘summary’ window and re-analyse (will need to re-threshold)
3. When batch is completed, copy the contents of the ‘summary’ window to Excel
4. You will take 4 measurements per tibia and output required is total area:


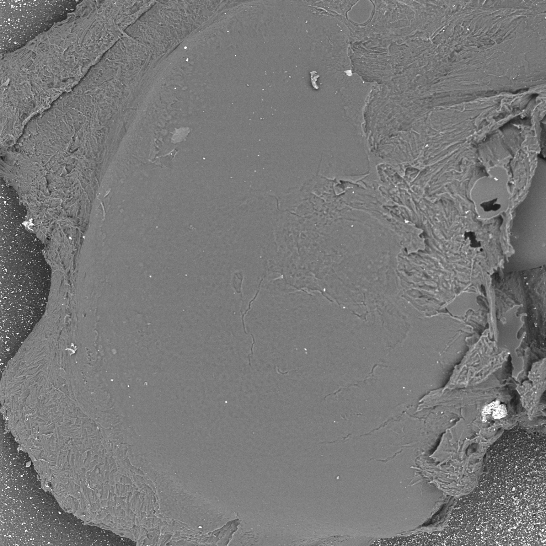


Original image


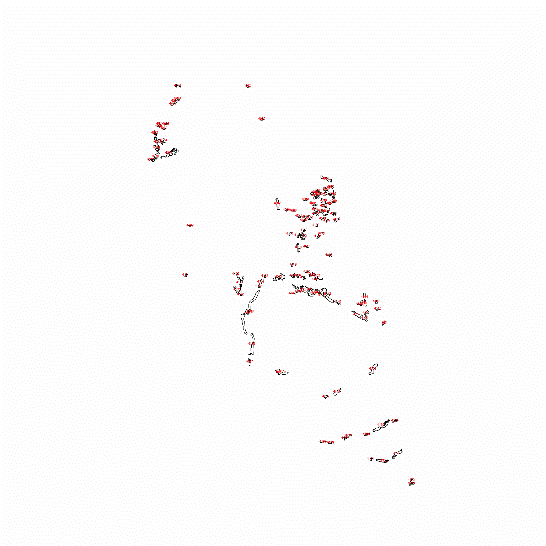


Damage mask (detected damage)

- 1. Whole plateau area (lateral tibial plateau)
  2. Damage area (lateral tibial plateau)
  3. Whole plateau area (medial tibial plateau)
  4. Damage area (medial tibial plateau)

**Appendix 2: Demo instructions for macro 7 (subchondral X-ray microradiography)**

1. Open the file **Demo_Data_Butterfield_macro_7.dcm** in ImageJ1.44
2. Find the modal grey level value of the steel standard (high intensity, bottom of image) by selecting a small amount and pressing ‘h’
3. Find the modal grey level of the plastic standard (low intensity, left of image) by selecting a small amount and pressing ‘h’
4. Find the minimum and maximum grey level in the whole image by pressing ‘h’
5. Install and run ***Macro 7***
   1. Input the modal values for steel, plastic, and min and max obtained
   2.
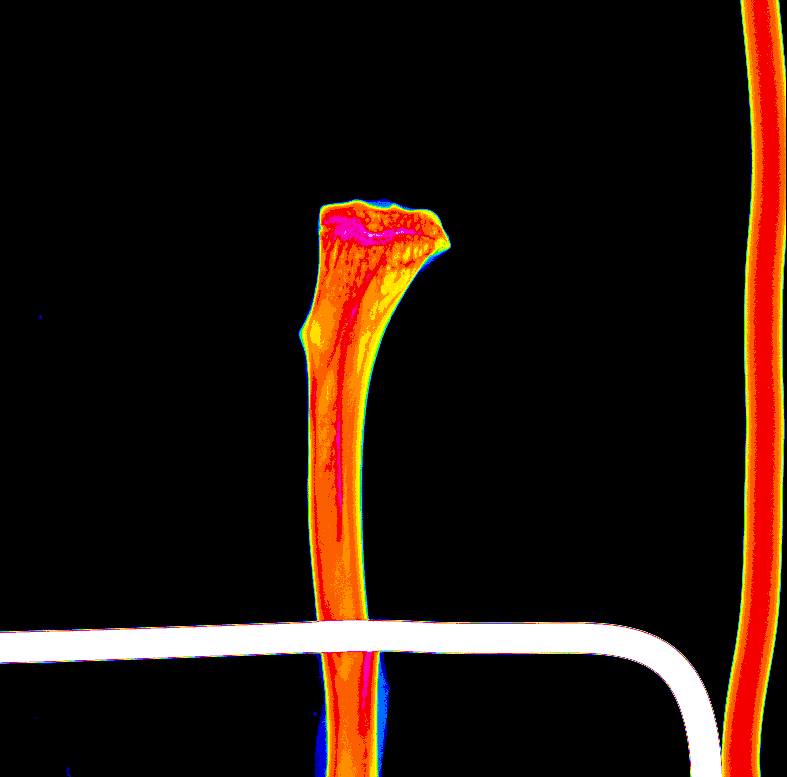
Save 16-colour image as tiff file for analysis


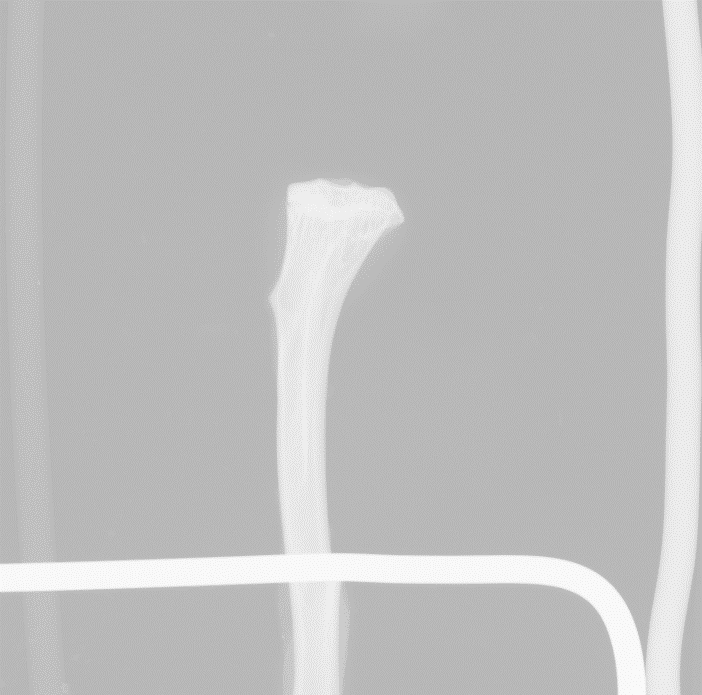


Plastic

Steel
