# Supplementary figures and images for: Accelerating functional gene discovery in osteoarthritis
Source: Nat Commun. 2021 Jan 20;12:467. doi: 10.1038/s41467-020-20761-5 (PMC7817695; doi:10.1038/s41467-020-20761-5)

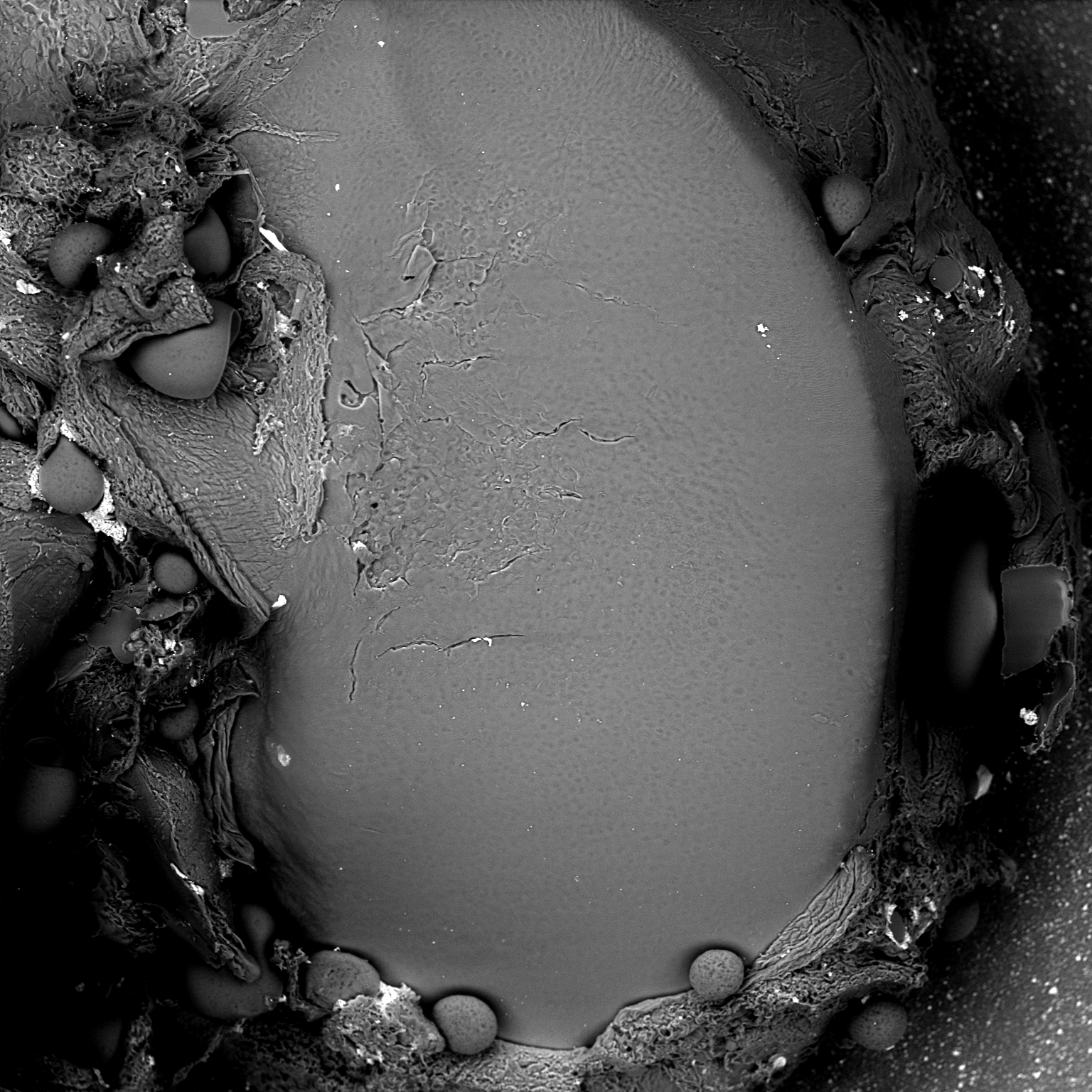

Supplement: Supplementary file 21 — Supplementary Software [file 41467_2020_20761_MOESM21_ESM.zip › Butterfield et al CODE AND SOFTWARE/Demo data/Demo_Data_Butterfield_macros1-6.tif]
