# Supplementary material for: Accelerating functional gene discovery in osteoarthritis
Source: Nat Commun. 2021 Jan 20;12:467. doi: 10.1038/s41467-020-20761-5 (PMC7817695; doi:10.1038/s41467-020-20761-5)
Supplement: Supplementary file 23 — Description of Additional Supplementary Files [file 41467_2020_20761_MOESM23_ESM.pdf]

Title: Supplementary Data 1

Description: Osteoarthritis Research Society International (OARSI), synovitis and osteophyte scoring in sham and destabilization of the medial meniscus (DMM)-operated limbs by histology. Source data are provided as a Source Data file.

Title: Supplementary Data 2

Description: Osteophyte scoring in sham and destabilization of the medial meniscus (DMM)-operated limbs by iodine contrast-enhanced micro-computerized tomography (ICEµCT) and joint surface replication (JSR). Source data are provided as a Source Data file.

Title: Supplementary Data 3

Description: Datasets for all joint phenotyping pipeline parameters in 100 16-week-old wild- type mice. Source data are provided as a Source Data file.

Title: Supplementary Data 4

Description: Joint phenotyping pipeline data from 50 randomly selected 16-week-old mutant mouse lines. Source data are provided as a Source Data file.

Title: Supplementary Data 5

Description: Statistical analysis of joint phenotyping pipeline data from 50 randomly selected 16-week-old mutant mouse lines

Title: Supplementary Data 6

Description: Spearman correlation matrix for statistically significant correlation coefficients (R-values, $P<0.05$) between joint phenotyping parameters. Source data are provided as a Source Data file.

Title: Supplementary Data 7

Description: Prioritization analysis of 25 mutant mouse lines with abnormal joint phenotypes at 16 weeks-of-age. Source data are provided as a Source Data file.

Title: Supplementary Data 8

Description: Osteophyte scoring in mutant mice by iodine contrast-enhanced micro-computerized tomography (ICEµCT) and joint surface replication (JSR)

Title: Supplementary Data 9

Description: Time required to perform Origins of Bone and Cartilage Disease (OBCD) joint phenotyping

Title: Supplementary Data 10

Description: Time required to perform Osteoarthritis Research Society International (OARSI) joint phenotyping

Title: Supplementary Data 11

Description: Cost analysis of Origins of Bone and Cartilage Disease (OBCD) versus Osteoarthritis Research Society International (OARSI) joint phenotyping

Title: Supplementary Data 12

Animal species, strain, source, sex and unique identifier or Research Resource Identifier

Title: Supplementary Data 13

Description: Determination of the volume of interest (VOI) for iodine contrast-enhanced micro-computerized tomography (ICEµCT) and effect of Lipiodol on subchondral bone parameters. Source data are provided as a Source Data file.

Title: Supplementary Data 14

Description: ImageJ macros for automated quantitation of joint surface replication (JSR) and subchondral X-ray microradiography (scXRM).

Title: Supplementary Data 15

Description: Repeatability of measurement of joint phenotyping parameters in 16-week-old wild type and mutant mice. Source data are provided as a Source Data file.

Title: Supplementary Data 16

Description: Correlation between phenotype magnitude and precision error. Source data are provided as a Source Data file.

Title: Supplementary Data 17

Description: *P*-values for validation of new methods with surgical provocation of osteoarthritis, age-related osteoarthritis and Dio2 polymorphism mice. Source data are provided as a Source Data file.

Title: Supplementary Data 18

Description: Power calculations. Source data are provided as a Source Data file.

Title: Supplementary Software

Description: Custom macros to automate image analysis using ImageJ are provided and are publicly available at https://github.com/Molendo/OBCD (Supplementary Data 14). Here we provide instructions for use (**Butterfield et al Code and Software submission.docx**) and demo data (**Demo data**) for automated quantitation of cartilage damage by joint surface replication (JSR) and quantitation of subchondral bone mineral content by subchondral X-ray microradiography.
